# Distinct Agents Induce Streptococcus mutans Cells with Altered Biofilm Formation Capacity

**DOI:** 10.1128/spectrum.00650-22

**Published:** 2022-07-11

**Authors:** Ana Carolina Urbano de Araujo Lopes, Carmélia Isabel Vitorino Lobo, Sabrina Marcela Ribeiro, Jaqueline da Silva Colin, Vanessa Coronato Nogueira Constantino, Matheus Mieli Canonici, Paula Aboud Barbugli, Marlise Inêz Klein

**Affiliations:** a Department of Dental Materials and Prosthodontics, São Paulo State University (Unesp), School of Dentistry, Araraquara, São Paulo, Brazil; Griffith University

**Keywords:** biofilms, extracellular matrix, *Streptococcus mutans*, dental caries, treatments, antimicrobial tolerance, antibiofilm agents, antimicrobial agents, persisters

## Abstract

Dental caries is a multifactorial biofilm- and sugar-dependent disease. This study investigated the influence of different agents on the induction of surviving Streptococcus mutans cells after successive treatment cycles and characterized the biofilms formed by these cells recovered posttreatment. The agents (with their main targets listed in parentheses) were compound 1771 (lipoteichoic acids), 4′ hydroxychalcone (exopolysaccharides), myricetin (exopolysaccharides), *tt*-farnesol (cytoplasmatic membrane), sodium fluoride (enolase—glycolysis), chlorhexidine (antimicrobial), and vehicle. Recovered cells from biofilms were generated from exposure to each agent during 10 cycles of consecutive treatments (modeled on a polystyrene plate bottom). The recovered cell counting was different for each agent. The recovered cells from each group were grown as biofilms on saliva-coated hydroxyapatite discs (culture medium with sucrose/starch). In S. mutans biofilms formed by cells recovered from biofilms previously exposed to compound 1771, 4′ hydroxychalcone, or myricetin, cells presented higher expression of the *16S rRNA, gyrA* (DNA replication and transcription)*, gtfB* (insoluble exopolysaccharides), and *eno* (enolase—glycolysis) genes and lower quantities of insoluble dry weight and insoluble exopolysaccharides than those derived from other agents. These findings were confirmed by the smaller biovolume of bacteria and/or exopolysaccharides and the biofilm distribution (coverage area). Moreover, preexposure to chlorhexidine increased exopolysaccharide production. Therefore, agents with different targets induce cells with distinct biofilm formation capacities, which is critical for developing formulations for biofilm control.

**IMPORTANCE** This article addresses the effect of distinct agents with distinct targets in the bacterial cell (cytoplasmatic membrane and glycolysis), the cell’s extracellular synthesis of exopolysaccharides that are important for cariogenic extracellular matrix construction and biofilm buildup in the generation of cells that persisted after treatment, and how these cells form biofilms *in vitro*. For example, if preexposure to an agent augments the production of virulence determinants, such as exopolysaccharides, its clinical value may be inadequate. Modification of biofilm formation capacity after exposure to agents is critical for the development of formulations for biofilm control to prevent caries, a ubiquitous disease associated with biofilm and diet.

## INTRODUCTION

Dental caries is a worldwide public health problem (1), and its prevention is critical for maintaining systemic and oral health. It is a multifactorial, biofilm- and sugar-dependent chronic disease. Dental caries is initially characterized by enamel demineralization because of shifts in metabolic activity and biofilm ecology driven by the frequent intake of sugars (2). Dental biofilm is a structured and organized microbial community. This biofilm’s formation is dynamic and dependent on the development of extracellular matrix, which is rich in exopolysaccharides (EPSs) (3, 4).

Streptococcus mutans has a prominent role among the species of the oral microbiota. Although not the predominant species in quantitative terms (5, 6), this species is acidogenic (able to produce acids from sugar fermentation) and aciduric (able to tolerate an acidic environment). However, its relevance is due to its unique ability to build an extracellular matrix. This species can produce and export exoenzymes such as glycosyltransferases (Gtfs) that can be part of the salivary pellicle and adsorb to microbial surfaces, leading to exopolysaccharide production *in situ* (3). Furthermore, as they act on different surfaces (teeth and microbial cells) and saliva, they allow adhesion and accumulation of other microorganisms on the teeth, contributing to the maturation of the biofilm.

In addition to exopolysaccharides, the extracellular matrix of cariogenic biofilms is also made up of extracellular DNA (eDNA) and lipoteichoic acids (LTA) (7). These interact with exopolysaccharides to form biofilms and affect S. mutans virulence (8, 9). Both eDNA and LTA influence the distribution of exopolysaccharides and the structural organization of microcolonies in the biofilm (8). Proteins are also part of S. mutans biofilms (10).

The conventional control of cariogenic biofilm is through its mechanical removal by toothbrushing and flossing (11). Fluoride associated with oral hygiene is used to prevent caries by promoting the control of dental surfaces’ demineralization (12). Chemotherapy with antimicrobial agents can be included, especially when the cariogenic challenge is high (e.g., in hospitalized patients and patients with psychomotor difficulties) (13). Thus, bioactive agents that affect the virulence and/or the ability of pathogenic microorganisms to develop biofilm are being searched for to control cariogenic biofilm as part of prevention strategies (14, 15).

However, there is a lack of knowledge on the degree of resistance, tolerance, and persistence of the oral microorganism in response to the chemotherapy agents used clinically and experimental agents. A close relationship between bacterial persistence and the recurrence of chronic diseases has been reported (16). Furthermore, persister cells (17, 18) have already been found in several cultures, including S. mutans (19, 20), which could influence the severity and recurrence of carious lesions.

Resistance is the inherited ability of bacteria to grow at high concentrations of an antibiotic. Bacterial drug resistance is a classic form of survival: it is the ability of microorganisms to grow even at high concentrations of antibiotics. Resistance is usually caused by hereditary mutants in which a biological mutation is often accompanied by a change in the genetic DNA sequence or chromosome (21, 22). Tolerance is the ability of a bacterial population to survive a certain time of exposure to bactericidal antibiotics, even at concentrations that far exceed the MIC (23). Persisters are subpopulations of cells in a dormant state with a phenotype of little or no growth (22). They have decelerated metabolic processes, with little or no cell wall production and protein synthesis (24). Therefore, they do not replicate, nor do they die in the presence of antimicrobial agents, thus presenting transient tolerance to several drugs (17). However, in the absence of antimicrobial agents, they return to the state of susceptibility, starting new bacterial growth and repopulating the environment (22, 25). Laboratory studies demonstrated that few persisters are formed in the initial exponential phase, but they significantly increase in half of the exponential growth phase (mid-log), reaching their maximum in the stationary growth phase (26). Moreover, the likelihood of persister cells depends on the mechanism of action of the antimicrobial agent. For example, if active growth of the bacterial target is not required, then bactericidal agents are less likely to result in persister cells.

Persistence is an example of nongenetic single-cell heterogeneity and may have a role in failing antimicrobial and antibiofilm chemotherapy treatments. Thus, this study investigated the influence of different agents with distinct targets on the generation of Streptococcus mutans cells that persisted after successive applications of agents (i.e., cells recovered posttreatment) and characterized the biofilms formed by these recovered cells. The agents used had known targets in S. mutans cells or metabolic activity associated with biofilm formation (14, 15). The rationale for the study is that, in the mouth, the topically applied agents (e.g., as mouthwash) are removed by the action of saliva, talking, and eating (i.e., clearance). Moreover, whether microbial cells will form the same biofilm formation capacity after agent removal is unknown. In other words, how oral microbial cells that were exposed to agents will behave and form biofilms when the use of these agents is stopped is undetermined. This knowledge is pertinent because of the clearance in the mouth of topically applied agents or the agents’ dilution by saliva, and usually agents are used for a specific period (e.g., chlorhexidine for 14 days). Thus, the approach employed to recover cells from biofilms after exposure was chosen to emulate the presence of a constant lower concentration in contact with biofilms that could be achieved when using a “smart” delivery system (e.g., nanocarriers that release drugs at pathogenic low pH) (27, 28).

## RESULTS

### Growth inhibition and killing curves.

**FIG 1:**
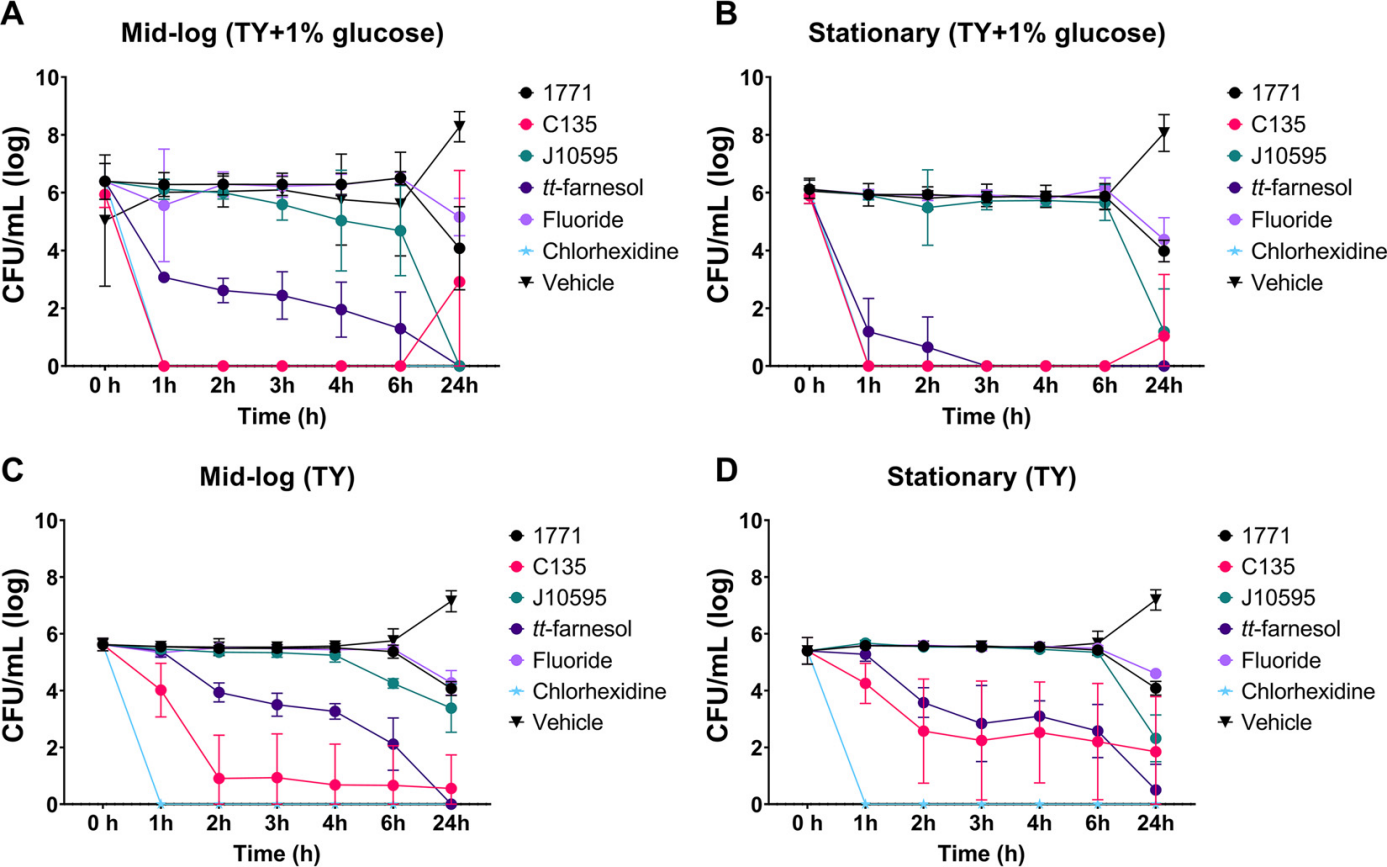
Streptococcus mutans growth inhibition (TY+1% glucose) and killing (TY) curves by agents incubated with cells at the mid-log (A, C) and stationary (B, D) growth phases. Each time is represented by the mean, and error bars are the standard deviation. 1771, compound 1771; C135, 4′ hydroxychalcone; J10595, myricetin; Fluoride, sodium fluoride; Vehicle, EtOH plus DMSO. The data without log10 transformation is presented in Table S1.

The levels of growth inhibition at the mid-log and stationary growth phases are shown in Fig. 1A and B, respectively. The vehicle control showed bacterial growth from 6 to 24 h in both settings. At the concentration normally recommended for biofilm control of 0.12%, the positive control, chlorhexidine, leads to 100% cell death after exposure for 1 h in both settings. The sodium fluoride (fluoride) control delayed growth and affected the viability of S. mutans between 6 and 24 h in both settings. Compared with the vehicle control, 4′ hydroxychalcone (C135) inhibited cell growth from 1 to 6 h; however, growth was observed from 6 to 24 h in both settings, being more pronounced in the mid-log setting. Both *tt*-farnesol and myricetin (J10595) inhibited cell growth over time in the mid-log setting. In contrast, *tt*-farnesol completely inhibited cell growth after 2 h of exposure, and myricetin was less effective in the stationary setting. Compound 1771 reduced cell viability between 6 and 24 h in both settings.

The killing curves at the mid-log and stationary growth phases are shown in Fig. 1C and D, respectively. Even in the absence of glucose, the cells incubated with the vehicle control grew in both settings, but less than in the presence of glucose (Fig. 1A and B). The positive control, chlorhexidine, killed all cells in both settings. Fluoride and compound 1771 killed cells from 6 to 24 h in both settings. *tt*-Farnesol killed cells gradually over time in both settings, while 4′ hydroxychalcone killed cells more efficiently in the mid-log setting than in the stationary setting. Also, 4′ hydroxychalcone was more efficient at growth inhibition in the presence of glucose (Fig. 1A and B) than in the absence of glucose (Fig. 1C and D). Finally, myricetin promoted gradual cell death from 4 to 24 h at the mid-log setting, but not as efficiently as in the presence of glucose.

### S. mutans cells recovered from biofilms posttreatment.

**FIG 2:**
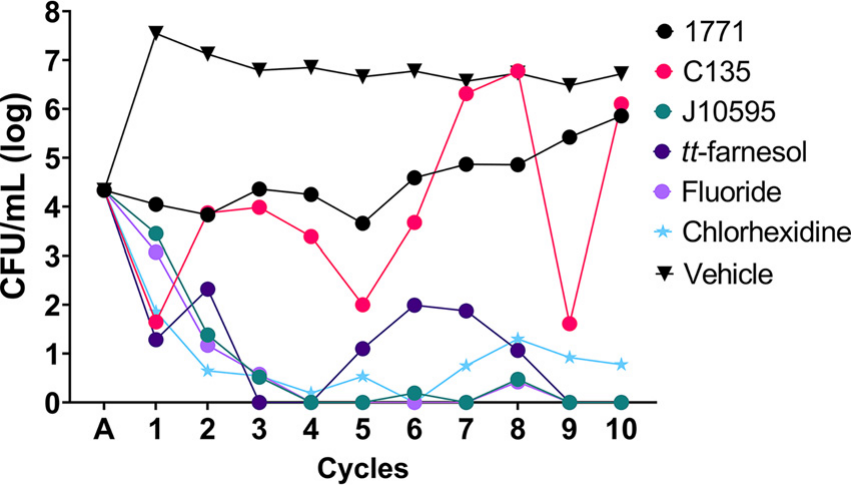
Recovered cell generation throughout 10 treatment cycles. Each point is represented by the mean. A, adhesion. The data without log10 transformation is presented in Table S3.

The profile of recovered cells (i.e., cells that persisted after exposure to distinct agents) is illustrated in Fig. 2 (see Table S2 in the supplemental material). Persistence of cells posttreatment with the vehicle was constant throughout the cycles. The positive control chlorhexidine reduced cell viability but did not result in permanent cell growth inhibition (absence of detection only in cycles 4 and 6). As for the sodium fluoride control, cells were no longer detected after four treatment cycles.

Among the drugs tested, compound 1771 presented similar behavior to the vehicle control: both showed a constant profile of colony detection throughout the cycles, without a marked reduction in cell viability. On the other hand, the group treated with 4′ hydroxychalcone presented a decrease in cell viability, with some increase in bacterial population through the cycles, especially in cycles 7, 8, and 10. Myricetin promoted a gradual decrease in cell viability, without colony recovery after three treatment cycles but with detection in the eighth cycle. *tt*-Farnesol manifested a variation between experiments, with a biphasic profile, in which there were periods of cell growth and death through the cycles. The selected cycles on each experimental occasion for biofilm formation are shown in Table 1.

**TABLE 1:** Selected cycles on each experimental occasion for biofilm development on saliva-coated hydroxyapatite discs

Treatment^*a*^	Selected cycle on:		
	Occasion 1	Occasion 2	Occasion 3
Compound 1771	C8	C8	C8
C135	C8	C8	C8
J10595	C1	C1	C3
*tt-*Farnesol	C2	C2	C2
Sodium fluoride	C1	C2	C1
Chlorhexidine	C1	C1	C3
Vehicle	C8	C8	C8

a C135, 4′ hydroxychalcone; J10595, myricetin; Vehicle, EtOH plus DMSO.

### Characterization of biofilms formed by S. mutans cells recovered posttreatment.

### (i) pH of the spent culture medium.

**FIG 3:**
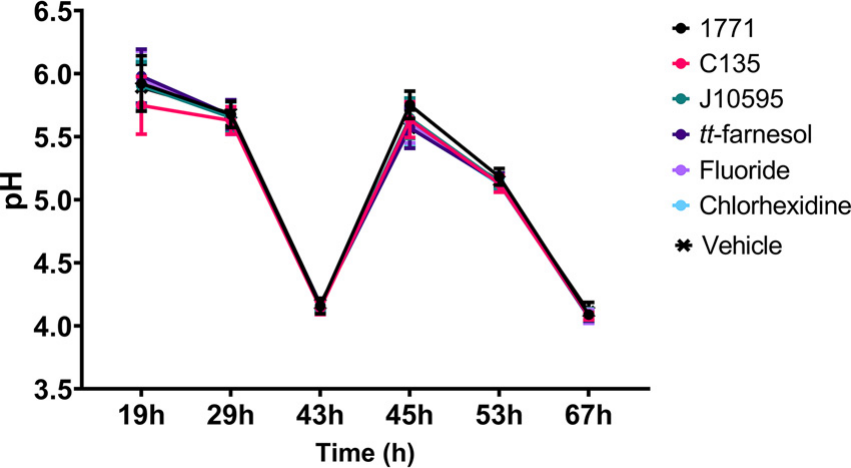
pH of spent culture media from biofilms formed on saliva-coated hydroxyapatite discs at distinct developmental phases. Each time is represented by the mean, and error bars are the standard deviation.

The pH of the spent medium was measured after changing the culture medium during the development of biofilms at 19, 29, 43, and 53 h and when the biofilms were removed for analyses at 45 and 67 h (Fig. 3). These values reflect acidogenicity, which measures the concentration of free hydrogen ions in the medium. However, they do not show whether acid concentration occurred at a specific location within the biofilm or in the interface between the biofilm and the substrate (hydroxyapatite disc).

The mean values for ages 43, 53, and 67 h are lower (or more acidic) than those at 19 h and are below 5.5—the pH value considered critical for demineralization of tooth enamel (29). However, there was a similarity in the pH values between the evaluated groups over time.

The culture medium used contained different sucrose and starch concentrations during different biofilm formation periods. Therefore, the pH values at 19, 29, 45, and 53 h represent the metabolization of tryptone-yeast extract broth (TY) plus 0.1% sucrose plus 20% saliva after incubation for 19, 10, 2, and 10 h, respectively. On the other hand, the pH values at 43 and 67 h represent the metabolization of TY plus 0.5% sucrose plus 1% starch plus 20% saliva after incubation for 14 h. Hence, the lower pH values were observed at 43 and 67 h.

### (ii) Gene expression.

**FIG 4:**
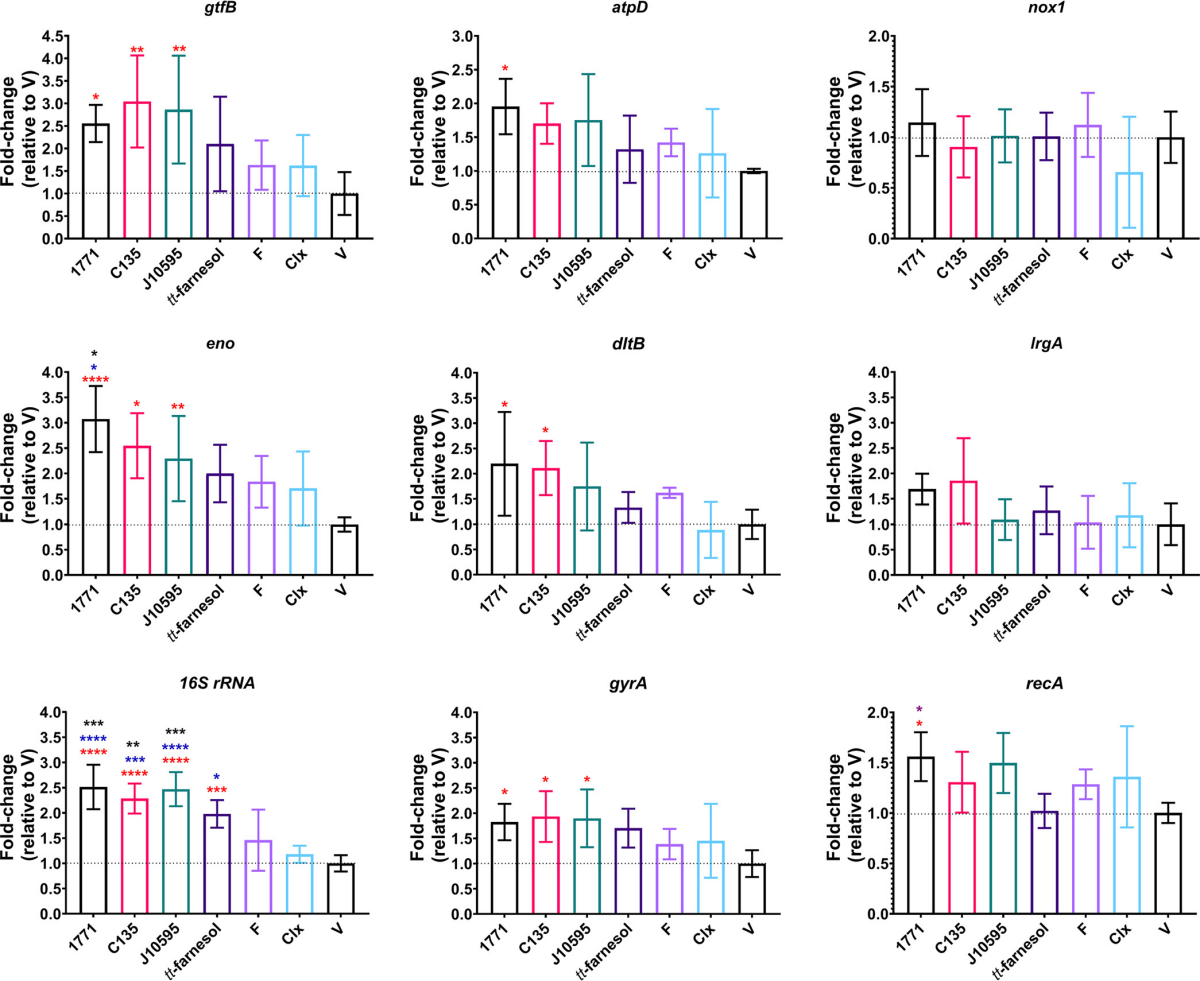
Streptococcus mutans gene expression in 45-h biofilms derived from cells recovered posttreatment. Data are the mean, and the error bars are the standard deviation. Multiple comparisons (i.e., all groups were compared to each other) were performed for all genes, and statistically significant comparisons are indicated with asterisks with distinct colors: black represents comparison with sodium fluoride, blue with chlorhexidine, red with the vehicle, and purple with *tt-*farnesol (only for *recA*). Thus, statistical difference is indicated as follows: *, *P* ≤ 0.05; **, *P* < 0.005; ***, *P* ≤ 0.0001; and ****, *P* ≤ 0.000 (one-way ANOVA followed by Tukey's test). Clx, chlorhexidine; V, vehicle.

The gene expression data obtained for S. mutans (*gtfB*, *atpD*, *nox1*, *eno*, *dltB*, *lrgA*, *16S rRNA, gyrA*, and *recA*) are illustrated in Fig. 4. In biofilms of cells recovered posttreatment, the *16S rRNA* gene presented higher expression for groups treated with compound 1771, 4′ hydroxychalcone, myricetin, and *tt*-farnesol than the groups treated with vehicle and chlorhexidine (one-way analysis of variance [ANOVA], *P* ≤ 0.0001, followed by Tukey’s test, *P* ≤ 0.0275). Also, the expression of *16S rRNA* was higher for the groups treated with compound 1771, 4′ hydroxychalcone, and myricetin compared to fluoride (*P* ≤ 0.0071).

For the *gtfB*, there was higher expression for the groups treated with compound 1771, 4′ hydroxychalcone, and myricetin compared to vehicle (one-way ANOVA, *P* = 0.0014, followed by Tukey's test, *P* ≤ 0.0390). For *eno* and *gyrA*, there was also a greater expression for these three groups (compound 1771, C135, and J10595) versus vehicle (one-way ANOVA, *P* ≤ 0.0111, followed by Tukey's test, *P* ≤ 0.0469). Also, the expression of *eno* was higher for compound 1771 than for fluoride and chlorhexidine (*P* ≤ 0.0224).

The genes *atpD*, *dltB*, and *recA* presented a similar expression profile; they all presented a higher expression for compound 1771 versus vehicle (one-way ANOVA, *P* ≤ 0.0196, followed by Tukey's test, *P* ≤ 0.0412). For *dltB*, C135 also presented higher expression than the vehicle (*P* = 0.0466). For *recA*, the group treated with compound 1771 showed higher expression than the group treated with *tt*-farnesol (*P* = 0.0404). However, *nox1* and *lrgA* did not present differences between groups.

### (iii) Viable counts of S. mutans, dry weight (insoluble biomass), and proteins (insoluble portion).

**FIG 5:**
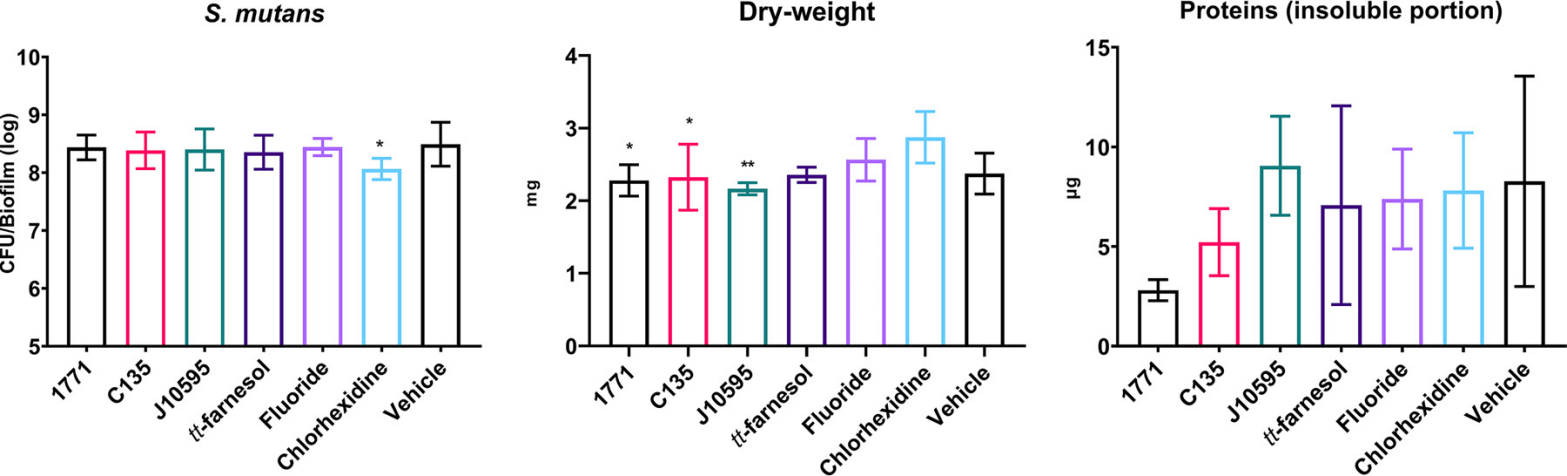
Viable counts of S. mutans, dry weight (insoluble biomass), and proteins (insoluble portion) of 67-h biofilms derived from cells recovered posttreatment. Data are represented by the mean, and error bars correspond to the standard deviation. Asterisks denote a difference between the specific groups versus chlorhexidine (*P* ≤ 0.05, one-way ANOVA followed by Tukey’s test). The CFU data without log10 transformation is presented in Table S4.

The quantification of viable S. mutans cells, dry weight, and proteins (at the insoluble portion) is shown in Fig. 5. The group treated with chlorhexidine presented a reduced count of the bacterial population compared to the others (one-way ANOVA, *P* = 0.0015, followed by Tukey's test, *P* = 0.0459). Moreover, the group treated with chlorhexidine showed higher dry weight than the groups treated with compound 1771, 4′ hydroxychalcone, and myricetin (one-way ANOVA, *P* = 0.0051, followed by Tukey's test, *P* = 0.0166, *P* = 0.0321, and *P* = 0.0044, respectively). The mean percentages of dry weight of the compound 1771, C135, and J10595 groups (versus chlorhexidine) were 20.65%, 19.14%, and 24.69%, respectively. Thus, although the biofilm formed by cells recovered after chlorhexidine treatment presented less of a microbial population count, the preexposure to this agent produced a biofilm with more dry weight.

The quantification of proteins (at the insoluble portion) showed no statistical differences between the groups (one-way ANOVA, *P* = 0.1824, followed by Tukey's test).

### (iv) Extracellular matrix components.

**FIG 6:**
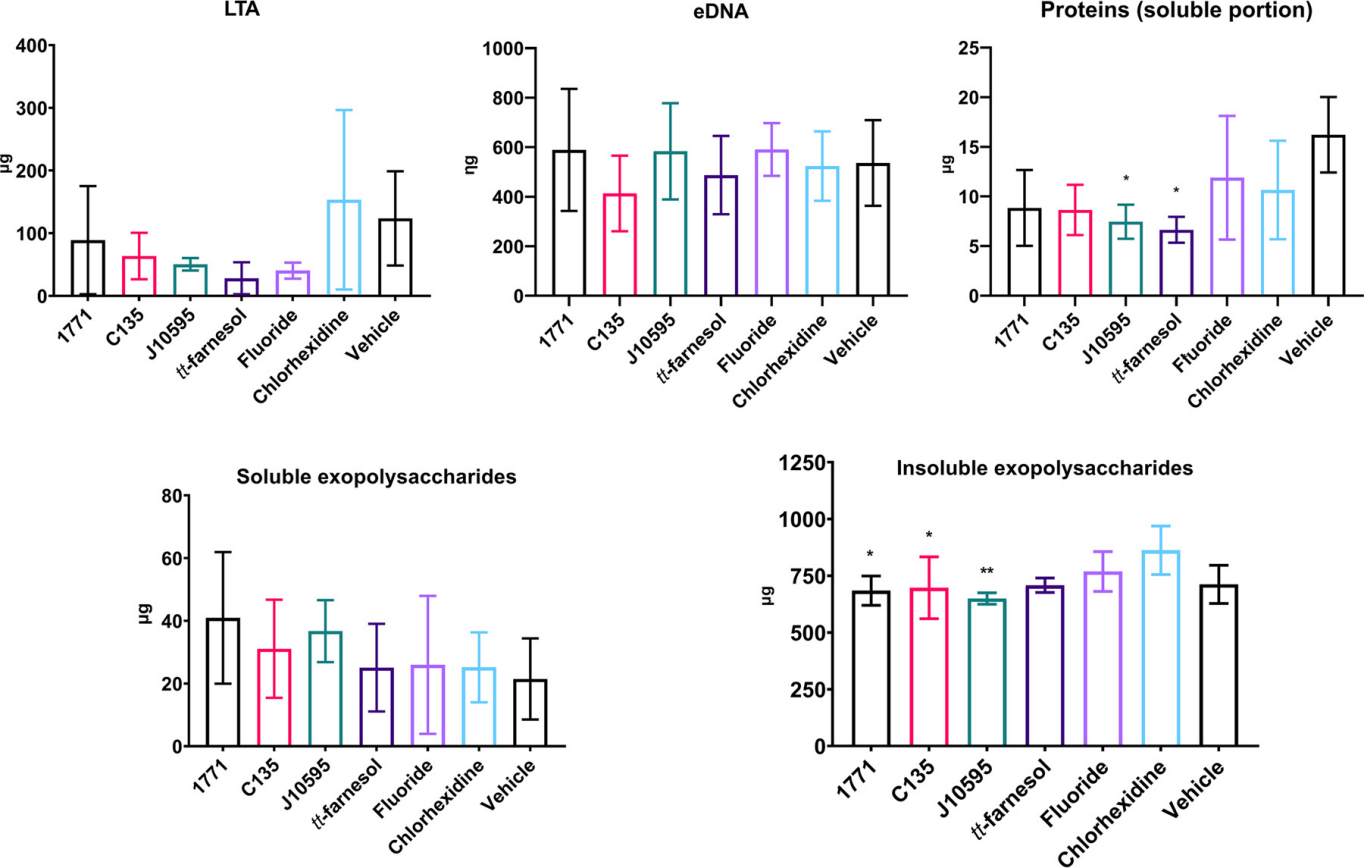
Extracellular matrix components of 67-h-old biofilms derived from cells recovered posttreatment. Graphs show quantification of LTA (lipoteichoic acids [micrograms]), eDNA (extracellular DNA [nanograms]), protein quantification (in the soluble portion [micrograms]), soluble exopolysaccharides (micrograms), and insoluble exopolysaccharides (micrograms). Data are represented by the mean, and the error bars correspond to the standard deviation. *, *P* ≤ 0.05; **, *P* ≤ 0.01 (one-way ANOVA followed by Tukey’s test).

The quantification of the extracellular matrix components is depicted in Fig. 6. Biofilms formed by cells recovered posttreatment with myricetin and *tt-*farnesol demonstrated smaller amounts of proteins (at the soluble portion) in their extracellular matrix than the vehicle (one-way ANOVA, *P* = 0.0124, followed by Tukey's test, *P* = 0.0265 and *P* = 0.0117, respectively).

For insoluble exopolysaccharides, smaller amounts were observed for the groups treated with compound 1771, 4′ hydroxychalcone, and myricetin versus the group treated with chlorhexidine (one-way ANOVA, *P* = 0.0052, followed by Tukey's test, *P* = 0.0168, *P* = 0.0317, and *P* = 0.0045, respectively). Thus the larger amount of insoluble exopolysaccharides in the biofilm formed by cells recovered after treatment with chlorhexidine may justify its smaller bacterial population since insoluble exopolysaccharides may have compromised cell dispersion during the sonication process (8). Furthermore, insoluble exopolysaccharides are the main component of the associated matrix for dry weight; a decrease in this component justifies the lower dry weight in groups treated with compound 1771, 4′ hydroxychalcone, and myricetin.

However, no significant differences were observed between the groups for eDNA (one-way ANOVA, *P* = 0.5453, followed by Tukey's test), soluble exopolysaccharides (one-way ANOVA, *P* = 0.2737, followed by Tukey's test), and LTA (one-way ANOVA, *P* = 0.9582, followed by Tukey's test).

### (v) Structure, biovolume, and percentage of coverage distribution of bacteria and exopolysaccharides.

**FIG 7:**
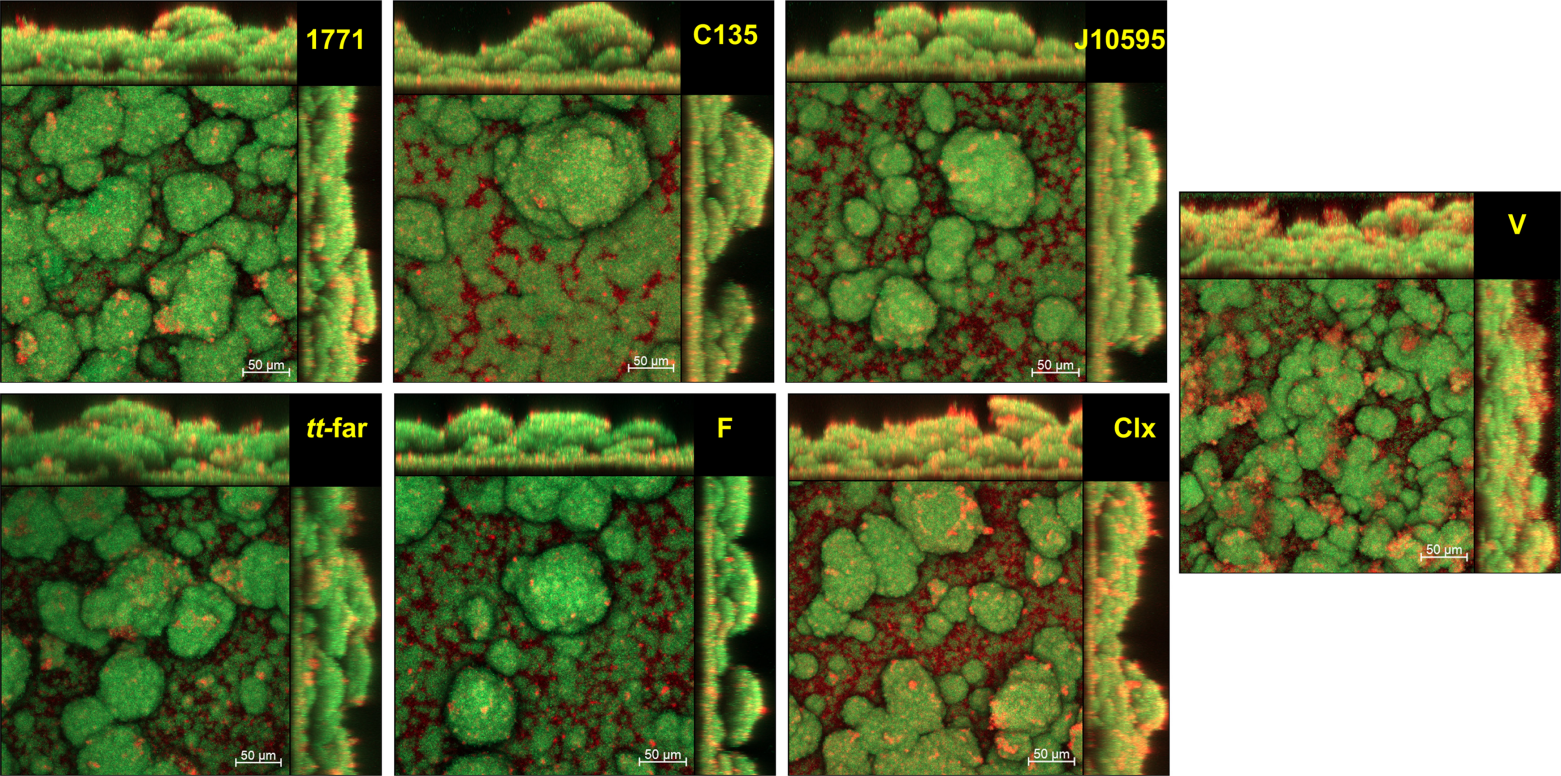
Representative images of biofilms formed by cells recovered posttreatment. The images are the overlay of the bacteria (labeled with SYTO9 and depicted in green) and exopolysaccharide content (labeled with Alexa Fluor 647 and depicted in red). *tt*-far, *tt*-farnesol; F, sodium fluoride.

**FIG 8:**
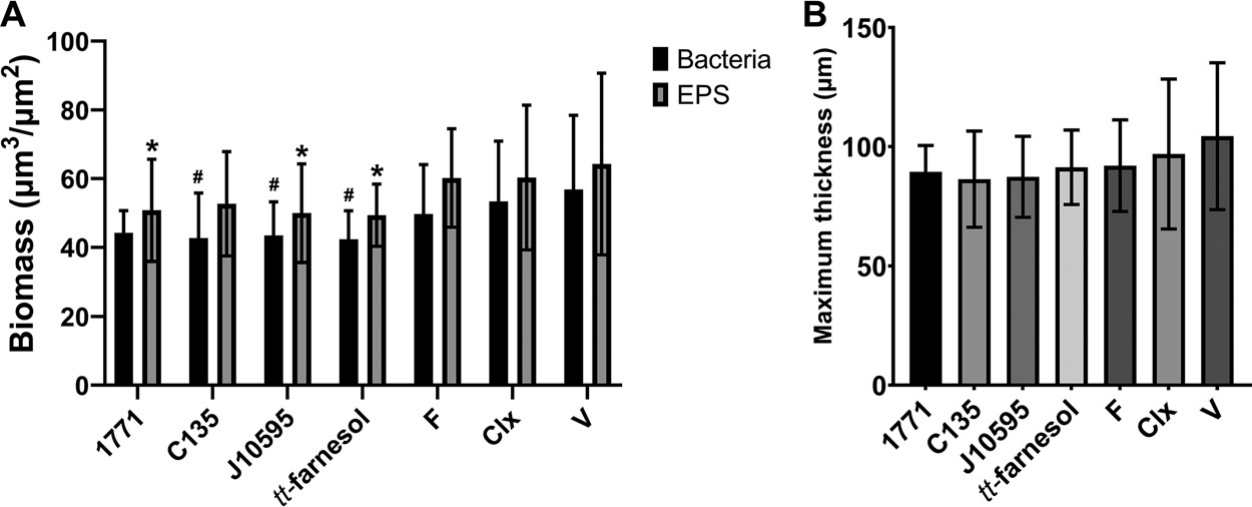
Biomass (A) and maximum thickness (B) of biofilms derived from cells recovered posttreatment. Data are represented by the mean, and error bars correspond to the standard deviation. In panel A, * denotes *P* ≤ 0.05 for the EPS (exopolysaccharide) component versus vehicle, and # denotes *P* ≤ 0.05 for the bacterial component versus vehicle (two-way ANOVA followed by Tukey’s test). 1771: compound 1771; C135: 4’ hydroxychalcone; J10595: myricetin; F: Sodium fluoride; Clx: Chlorhexidine; V: Vehicle (EtOH + DMSO).

Representative images of the three-dimensional (3D) structure of biofilms are shown in Fig. 7. The biomass or biovolume and maximum thickness are depicted in Fig. 8. The images indicate that biofilms derived from cells recovered posttreatment with compound 1771, myricetin, and sodium fluoride have smaller biofilm. In contrast, biofilms formed by cells recovered posttreatment with 4′ hydroxychalcone, myricetin, and chlorhexidine present more widely spaced clusters. These observations suggest that the treatments with different targets against biofilm components (cells or matrix composition) affect the 3D structure of biofilms derived from cells recovered posttreatment.

The biofilms formed by cells recovered after treatment with 4′ hydroxychalcone, myricetin, and *tt*-farnesol had a smaller amount of the bacterial component than the vehicle (two-way ANOVA, *P* = 0.0009, followed by Tukey's test, *P* = 0.0152, *P* = 0.0307, and *P* = 0.0136, respectively). Biofilms formed by cells recovered after treatment with compound 1771, myricetin, and *tt*-farnesol presented smaller amounts of exopolysaccharides than the vehicle (*P* = 0.0293, *P* = 0.0159, and *P* = 0.0099, respectively). The biofilms presented similar thicknesses (Fig. 8B) (one-way ANOVA, *P* = 0.1876, followed by Tukey's test).

**FIG 9:**
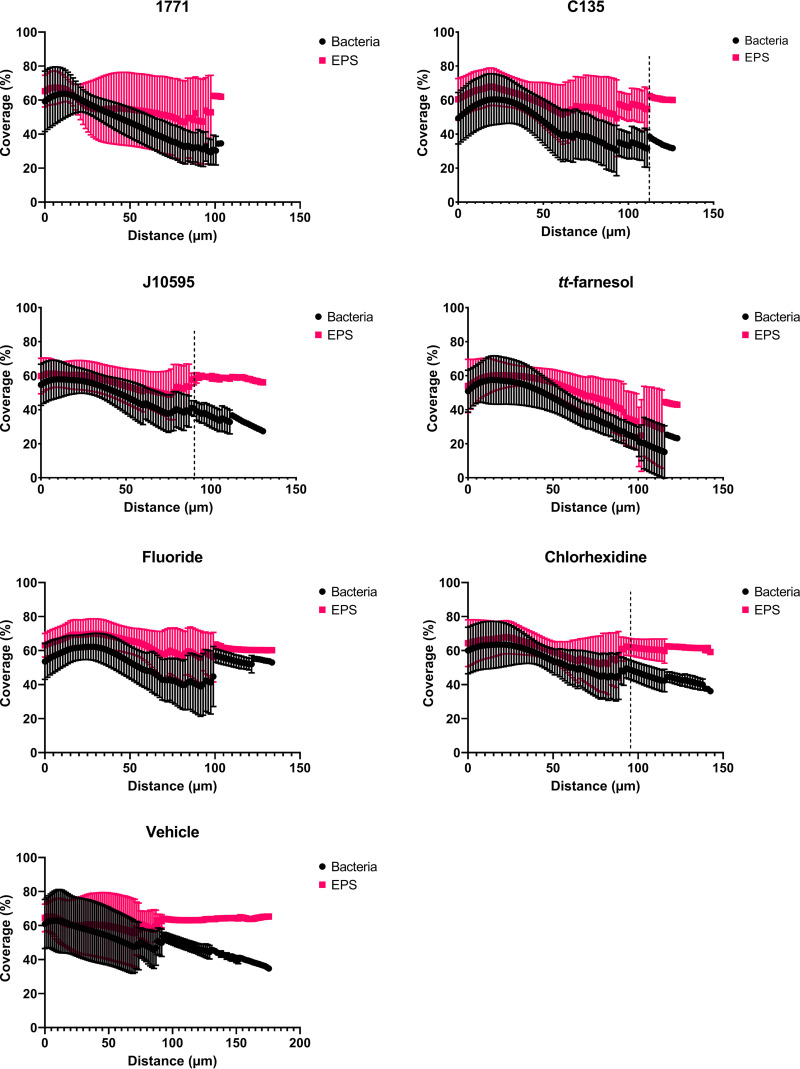
Percentage of coverage area of biofilms derived from cells recovered posttreatment. The data are represented by the mean and the standard deviation. EPS: exopolysaccharides. 1771: compound 1771; C135: 4’ hydroxychalcone; J10595: myricetin.

Regarding the percentage of coverage per area (Fig. 9), from the biofilm-hydroxyapatite disc interface to the surface, the biofilm formed by the vehicle-treated group presented the greatest thickness (as observed in Fig. 8B), with larger occupied areas (as also observed in the images in Fig. 7). The biofilms formed by groups treated with 4′ hydroxychalcone and sodium fluoride showed a larger area occupied by exopolysaccharides throughout the formed biofilm. Also, the group treated with 4′ hydroxychalcone presented a significant overlap of the exopolysaccharides over the bacterial component from about 115 μm onwards. Biofilms from groups treated with compound 1771 and *tt*-farnesol had similar proportions of areas occupied by exopolysaccharides and bacteria. Also, the biofilms from groups treated with myricetin and chlorhexidine presented similar ratios of exopolysaccharides to bacteria until ≅90 μm, while above this level, there was a predominance of exopolysaccharides.

## DISCUSSION

The focus of this study was to assess the influence of selected agents on the generation of S. mutans cells that persisted in biofilms posttreatment and characterize the biofilm formed by these cells in the absence of agents. However, initially, the action of agents on microbial viability was evaluated using planktonic culture at two growth phases because microbial cells are at different stages of growth within a biofilm (21). The effects on S. mutans growth and survival differed for different agents. Agents with distinct targets yielded different profiles of recovered cells in biofilms exposed to them. Moreover, the biofilms formed by these recovered cells in the absence of agents presented small differences in biofilm composition and organization, especially for insoluble exopolysaccharides, a virulence determinant (3).

The growth inhibition and killing profiles between planktonic growth phases differed slightly for the agents tested in the presence and absence of glucose. *tt*-Farnesol was more effective in the stationary phase and the second most effective agent in the mid-log growth phase. As *tt*-farnesol targets the cell membrane (14), its effectiveness in the stationary phase can be justified due to reduced metabolism, where cells need more time to start cell division, making the membrane more susceptible. This hypothesis also supports the greater effectiveness of sodium fluoride in the stationary growth phase because it targets glycolysis (30). On the other hand, myricetin, which mainly inhibits glucan synthesis and matrix development (15), promoted cell death in 24 h in the mid-log phase and reduced viability by ≅3 logs in the stationary phase. Furthermore, 4′ hydroxychalcone showed similar behavior in both phases in the absence of glucose. It inhibited bacterial growth from 1 to 6 h but allowed significant growth in 24 h, as previously observed (31). However, in the absence of glucose, 4′ hydroxychalcone was less effective in S. mutans viability because cells were detected in cultures at both growth phases treated. Hydroxychalcones inhibit S. mutans glycosyltransferase activity that hinders exopolysaccharide formation (32), but how these molecules affect cell growth is unknown. Future studies should uncover the mechanisms of action of hydroxychalcones associated with S. mutans death. Therefore, the stationary phase could be the critical target to eradicate S. mutans cells that persist posttreatment by blocking different metabolic pathways.

Chlorhexidine inhibited cell growth within 1 h of incubation for both planktonic growth phases, with and without glucose. However, despite its well-established effectiveness, it suppresses the oral microbiota (33), eliminating oral bacteria capable of converting nitrate to nitrite, which appears to be associated with an increase in systolic blood pressure (34, 35), since nitrate is absorbed in the gastrointestinal tract, reoxidized to nitric oxide and nitrate in the blood, and secreted in the saliva, a process known as the enterosalivary circulation of nitrate. Thus, maintaining a symbiotic oral microbiota contributes to general human health through the enterosalivary route of nitrate-nitrite-nitric oxide (34, 36). Therefore, chlorhexidine cannot be used for a continuous and prolonged period (37). In addition, it has known oral side effects, such as staining of teeth and restorations, mucosal peeling and ulcerations, temporary loss of taste, and burning in the mouth (38, 39), in addition to the possibility of bacterial recolonization (40).

S. mutans biofilms treated with distinct agents demonstrated that the bacterium persistence posttreatment could be linked to the known mechanisms of action of tested agents. The profiles of recovered cells posttreatment differed for each agent tested. As cells enter a dormant state, with little or no cell wall production (22), agents targeting the membrane or cell wall do not affect the cells’ metabolism, as observed for compound 1771 and 4′ hydroxychalcone. This behavior allows cells to remain constant throughout the treatment cycles (17). On the other hand, agents targeting glucan production and glycolysis had a more pronounced effect on the recovery of cells posttreatment, as observed for myricetin and sodium fluoride. The profile for *tt*-farnesol seems to be more related to the concentration used, in which it generated a biphasic cell recovery curve.

Taking into consideration that insoluble exopolysaccharide synthesis (glucans) interferes with the efficacy of biofilm control (41), a Δ*gtfB* strain was also used to detect the recovered cells posttreatment using the same agents and controls (under the same conditions used for parental strain UA159). 4′ hydroxychalcone, *tt*-farnesol, and chlorhexidine killed all cells (no colonies were detected after cycle 1), compound 1771 and myricetin yielded colonies in cycle 1, fluoride yielded them up to cycle 2, while vehicle yielded colonies in all treatment cycles (see Fig. S3 in the supplemental material).

After removing agents and under favorable development conditions, cells that are not killed can return to the state of susceptibility and repopulate the environment (22, 25). Therefore, the biofilms formed by the cells recovered posttreatment with distinct agents were analyzed. In an oral environment, even if a correct toothbrushing is performed, microbial cells may not be removed entirely, either because they are in the innermost layers of the biofilm or because they find themselves in unfavorable niches for mechanical removal. Thus, these cells can develop tolerance mechanisms by being subjected to constant doses of antimicrobial and/or antibiofilm agents.

At the same biofilm age, the biofilms derived from S. mutans cells recovered posttreatment with compound 1771, 4′ hydroxychalcone, and myricetin were at an earlier developmental phase than the others. This occurred because they present a higher gene expression in *16S rRNA* genes (which encode the smallest subunit of the ribosomes, which are the intracellular “protein factories”), *gtfB* (which encodes the exoenzyme GtfB, responsible for the synthesis of insoluble glucans), *eno* (which encodes the enolase enzyme of the glycolytic pathway), and *gyrA* (which encodes the DNA gyrase subunit A, involved in DNA replication and transcription), indicating that there was greater cell division activity, exopolysaccharide production, and glycolytic activity in these groups. Still, these groups had fewer insoluble exopolysaccharides in the matrix, the main component related to dry weight, which also supports its reduction. Finally, they had smaller amounts of exopolysaccharides and/or bacterial components, as demonstrated by confocal laser scanning microscopy (CLSM). A controversial aspect is that the numerical changes that presented statistical differences may or may not have a biological significance. However, we do not have an established cutoff to determine when a specific difference is or not biologically relevant in *in vitro* biofilms.

Although the pH of the spent medium during the growth of biofilms has shown similar values for the groups evaluated, differences may have occurred within the microcolonies and at the interface between microcolonies (biofilm) and the hydroxyapatite surface (substrate). Considering that the expression analysis was performed for all cells and not for cells located in specific parts of the biofilm, the higher expression of *atpD* for the group treated with compound 1771 could be because the cells (or at least part of them) needed to react to intracellular acidification (cytoplasmic) caused by the increased glycolytic activity (represented by the increased expression of *eno*). This milieu acidification led to the expression of the *atpD* gene, which encodes a functional subunit of the proton pump (F-ATPase, encoded by the genes of the *atpCDGAH*F operon, of which *atpD* shows the highest expression [42]) so that the cells could tolerate the acid being produced by themselves.

Furthermore, mean expression values of *atpD* for the biofilms in groups treated with 4′ hydroxychalcone and myricetin were higher and close to that of the group treated with compound 1771 (but without a statistical difference), compatible with the higher expression of *eno*. The response of S. mutans to environmental acidification also makes this microorganism more tolerant to oxidative stress, especially via an NADH oxidase encoded by *nox1* (43). Here, *nox1* expression showed similar mean values between all groups; therefore, this metabolic pathway showed no difference in biofilms derived from cells recovered posttreatment by different agents. It is noteworthy that the increased expression of *dltB* (a gene of the *dltABCD* operon, which encodes a membrane transport protein for d-alanine for the process of incorporation of d-alanine in lipoteichoic acids [44]) for compound 1771 may reflect a possible action of compound 1771 in the cytoplasmic membrane where DltB is located. In addition, the higher expression of *dltB* for compound 1771 could be because if this agent hinders the synthesis of teichoic acids by inhibiting the LTA synthase, increasing *dltB* could maximize the d-alanization of the fewer units in the cell wall. Both hypotheses need further confirmation with future research. Also, the mean expression of *lrgA* (a gene of the *lrgAB* operon), associated with cytoplasmic membrane remodeling, which is important in cell division, and cell lysis that releases eDNA (45, 46) were higher for compound 1771 and 4′ hydroxychalcone but did not differ statistically from the other groups. However, whether the influence of these agents on phenotypic and genotypic characteristics on cells and biofilms formed by these cells would be maintained for long periods after the removal of agents is unknown (47). In addition, whether these agents promote epigenetic changes deserves further investigation.

Thus, despite not being bactericidal agents, these compounds (1771, 4′ hydroxychalcone, and myricetin) affect cell viability as they target essential components of the bacterium, hindering its biofilm development (15). Myricetin and some chalcones affect bacterium F-ATPase activity, glycolysis, and glucan synthesis (15, 48). The glucan deficit compromises the microbial adhesion capacity (49, 50), hindering cell grouping, structural integrity, and 3D organization, impairing microcolony verticalization and expansion (51). Therefore, by directly or indirectly affecting the matrix, these agents can make the biofilm environment more susceptible to the action of antimicrobials (52), allowing the return to isolated forms so that their control is easier to occur.

The biofilms formed by cells derived after treatment with *tt*-farnesol, sodium fluoride, and chlorhexidine were more “mature.” Regarding the biofilm formed by cells after treatment with *tt*-farnesol, the proteins present in the soluble portion (matrix) may have been affected, impairing anchoring mechanisms and not allowing microbial aggregation, which would also reduce the area occupied by the microorganisms. As for sodium fluoride, although few cells were recovered posttreatment, when they form a biofilm on the hydroxyapatite surface, it is thick and has a significant coverage area on the surface where it grows. Despite chlorhexidine’s well-established antimicrobial efficacy, it produces cells that persist after treatments, and these recovered cells, in the absence of this antimicrobial (22, 25), form thicker biofilms with larger amounts of dry weight and insoluble exopolysaccharides (versus other agents and vehicle control). These factors can explain the reduced microbial population in this biofilm due to the difficulty in dispersing the cells during the sonication process; because the decrease of insoluble exopolysaccharides increases the S. mutans CFU count for a Δ*gtfB* strain (8).

This characteristic of biofilm maturity is associated with a more cohesive and “impenetrable” matrix (with reduced diffusion of substances in and out of biofilms) (3), with a predominance of insoluble glucans (29), which contribute to the creation of less permeable microniches, acidifying the medium (3). Furthermore, they render the biofilm more viscoelastic, hindering its removal by mechanical means (7) and facilitating surface demineralization.

Therefore, agents with different targets originate in S. mutans cells that were recovered posttreatment and presented altered biofilm formation capacity, which is important in developing formulations to control cariogenic biofilm. Thus, the active compounds in these formulations must not (i) trigger cells that persist after treatments, which will form new biofilms (when they are dislodged and move to other surfaces susceptible to colonization) with characteristics associated with virulence, such as a large amount of insoluble exopolysaccharides that build large and robust microcolonies, which can maintain the acidified environment at the biofilm-tooth interface and cause demineralization (3, 53) or (ii) affect the oral microbiota to influence both oral and systemic health.

In summary, in planktonic cultures, S. mutans growth inhibition (survival curves) demonstrated a distinct effect on cell viability, depending on the agent present. The persistence profile of S. mutans cells in biofilms differed for each agent evaluated over time, probably due to the different targets (mechanism of action on cells or extracellular matrix synthesis and organization). Moreover, the biofilms formed by S. mutans cells recovered after treatment with compound 1771, 4′ hydroxychalcone, and myricetin were at an earlier developmental phase than the others, while the groups treated with *tt-*farnesol and the controls sodium fluoride, chlorhexidine gluconate, and vehicle were at a more mature developmental phase. These characteristics of compound 1771, 4′ hydroxychalcone, and myricetin can reduce biofilm virulence in the absence of agents because the biofilms formed by cells preexposed to these three agents presented a tridimensional structure and composition with less insoluble exopolysaccharides. This trait means that these biofilms would have a greater susceptibility to the penetration of antimicrobial agents and mechanical removal.

## MATERIALS AND METHODS

### Experimental design.

An *in vitro* study with Streptococcus mutans was conducted to evaluate the influence of different agents on the generation of S. mutans cells that persisted after successive treatment cycles (i.e., cells recovered posttreatment) and characterize the biofilms formed by these cells. Seven treatments (agents and controls) were selected: compound 1771 (an inhibitor of LTA synthase) (48, 54), 4′ hydroxychalcone (which decreases Gtfs’ activity and exopolysaccharide synthesis) (32), myricetin (a flavonoid that hinders exopolysaccharide synthesis, acidogenicity, and acidurance) (15), *tt*-farnesol (a sesquiterpene associated with acidogenicity and acidurance) (14), sodium fluoride (a teeth demineralization prevention control associated with enolase in the glycolytic pathway) (12, 30), chlorhexidine (a broad-spectrum antimicrobial used as a positive control) (33), and vehicle (ethanol [EtOH] and dimethyl sulfoxide [DMSO]). All agents were prepared using the same vehicle.

Initially, killing curves (55) were assessed in planktonic culture in two growth phases—the middle of the exponential and the stationary phases—in culture medium with glucose (to assess growth inhibition) and without glucose (to assess biocidal effect). Then, biofilms were treated to induce cells that persisted with exposure to different agents in successive cycles. Finally, the recovered cells posttreatment were grown as biofilms in the absence of agents on saliva-coated hydroxyapatite (sHA) discs (mimetic of tooth enamel). These biofilms were characterized to verify whether the cells changed their biofilm formation phenotype after being exposed to distinct agents. This study was approved by the Institutional Ethics Committee (CAAE no. 31717914.3.0000.5416).

Experiments were performed on three occasions, in duplicate (six replicates, *n* = 3), for each experimental step described below. Data were analyzed according to the experimental design of the study.

### Test agents.

The following agents were used: compound 1771 [(5-phenyl-1,3,4-oxadiazol-2-yl)carbamoyl]methyl 2-{naphtho[2,1-b]furan-1-yl}acetate) (UkrOrgSynthesis, Ltd., catalog no. PB25353228; purity not available), 4′ hydroxychalcone (C135) [(2E)-1-(4-hydroxyphenyl)-3-phenylprop-2-en-1-one) (AK Scientific, Inc., catalog no. C135; 98% purity), myricetin (J10595) [3,5,7-trihydroxy-2-(3,4,5-trihydroxyphenyl)-4H-chromen-4-one] (AK Scientific, Inc., catalog no. J10595; 95% purity), *tt-*farnesol [(E,E)-3,7,11-trimethyl-2,6,10-dodecatrien-1-ol, *trans*,*trans*-3,7,11-trimethyl-2,6,10-dodecatrien-1-ol] (Sigma-Aldrich Co., St. Louis, MO, USA, catalog no. 46193; 96% purity)], sodium fluoride (Sigma-Aldrich, catalog no. 71519), chlorhexidine digluconate solution (Sigma-Aldrich, catalog no. C9394), and diluent vehicle, consisting of 7% ethanol (EtOH) (Sigma-Aldrich, catalog no. E7023) and 1.25% dimethyl sulfoxide (DMSO) (Sigma-Aldrich, catalog no. D8418).

For the killing curves, the concentrations used for each agent were 3.906 μg/mL (compound 1771), 250 μg/mL (4′ hydroxychalcone), 500 μg/mL (myricetin), 125 μg/mL (*tt*-farnesol), 250 ppm (sodium fluoride), and 0.12% (chlorhexidine). The concentrations of compound 1771, 4′ hydroxychalcone, myricetin, and *tt*-farnesol were selected based on the antimicrobial activity data previously tested (Table 2) (31, 56). Sodium fluoride and chlorhexidine concentrations were determined based on the concentrations usually found in commercial mouthwashes (57).

**TABLE 2:** Agents and their concentrations used for treatments for planktonic cultures and biofilms assays

Agent	Concn of agent used for:	
	Killing curves (planktonic cultures)^*a*^	Recovered cells posttreatment (biofilms)^*b*^
Compound 1771, μg/mL	3.906*	3.906
4′ hydroxychalcone (C135), μg/mL	250**	125
Myricetin (J10595), μg/mL	500**	250
*tt*-farnesol, μg/mL	125†	62.5^*c*^
Sodium fluoride, ppm	250	125
Chlorhexidine digluconate, %	0.12	0.0012
Vehicle	7% ethanol + 1.25% DMSO	7% ethanol + 1.25% DMSO

a The concentrations of compound 1771, C135, J10595, and *tt*-farnesol were selected based on antimicrobial activity data tested previously, where symbols correspond to specific concentrations: *, 3 × MIC (31); **, IC_50_ (50% reduction of CFU-per-milliliter counts versus the vehicle control [56]); †, 2 × IC_50_ (56). The concentrations of chlorhexidine and sodium fluoride were those found in mouthwashes (57).

b The concentrations to generate cells that could be recovered after treatments were selected based on previous studies (31, 56), and pilot tests were performed to make sure the concentrations were adequate (i.e., it was necessary that cells could be recovered at least after the first treatment cycle as colonies grown on agar plates).

c A lower concentration (32.25 μg/mL) did not affect the viability of the cell in biofilms (data from a pilot test [not shown]).

For the cells recovered posttreatment of biofilms, the concentrations used were 3.906 μg/mL (compound 1771), 125 μg/mL (4′ hydroxychalcone), 250 μg/mL (myricetin), 62.5 μg/mL (*tt-*farnesol), 125 ppm (sodium fluoride), and 0.0012% (chlorhexidine). The concentrations to recover cells posttreatment were initially selected based on previous studies (Table 2) (31, 56). Pilot trials were carried out to adjust these concentrations because it was necessary to recover colonies at least after the first treatment cycle.

### Microbial strain and growth conditions.

S. mutans UA159 (ATCC 700610) strain stocks were stored at −80°C and then plated on blood agar plates (5% sheep’s blood; Laborclin, Pinhais, PR, Brazil) at 37°C, in 5% CO_2_ for 48 h (Steri-Cult; Thermo Scientific, Waltham, MA, USA). Starter cultures were prepared by inoculating 5 colonies in tryptone-yeast extract broth (TY; 2.5% [wt/vol] tryptone, 1.5% [wt/vol] yeast extract [pH 7.0]) (Becton Dickinson and Company, Sparks, MD, USA) containing 1% (wt/vol) glucose (Synth, Diadema, SP, Brazil), followed by incubation for 16 h (37°C, 5% CO_2_). The starters were diluted 1:20 in fresh TY plus 1% glucose and incubated until the mid-log (optical density at 540 nm [OD_540_]) of 0.847 ± 0.273 and stationary (OD_540_ of 2.910 ± 0.030) (measured with a spectrophotometer from Kasvi, Beijing, China) growth phases for the killing curves. Cultures at the mid-log growth phase were also used to generate cells that would persist after treatment of biofilms.

For the formation of biofilms on saliva-coated hydroxyapatite discs, the cells recovered posttreatment with each agent on each different experimental occasion were used. The starter cultures were prepared by inoculating 10 μL of each stock (a colony stored in 50% [vol/vol] glycerol at −80°C) (Synth, Diadema, SP, Brazil) in fresh TY plus 1% glucose, followed by incubation for 16 h (37°C, 5% CO_2_). Then, the starter cultures were diluted at 1:20 in fresh TY plus 1% glucose and incubated until mid-log phase.

### Growth inhibition and killing curves.

Killing curves monitor bacterial growth and death over a wide range of antimicrobial concentrations and assess the effect of antimicrobials over time (58, 59). Although some of the agents are not antimicrobials *per se* because they target the extracellular matrix buildup, the killing curves were evaluated for the mid-log and stationary phases of bacterial growth using TY plus 1% glucose (growth inhibition) and TY (biocidal activity). First, the inoculum of the bacterial strain was prepared by adding 14.6 μL of the mid-log or stationary cultures to a Falcon tube containing 9.9854 mL TY plus 1% glucose or TY without glucose. This dilution was performed to standardize the culture concentration at 1 × 10^6^ CFU/mL. The culture was homogenized, and 300-μL aliquots were distributed to each well of a 48-well polystyrene microplate (Kasvi, Beijing, China) containing the treatments. Two replicates (wells) were prepared with the inoculum and one replicate (well) without the inoculum. The latter served as a parameter for the turbidity of treatments (control for visual observation of bacterial growth). An aliquot of the inoculum used in each experiment was plated to determine the amount of CFU per milliliter at time 0 h (before starting the incubation and adding treatments).

After incubation for 1, 2, 3, 4, 6, and 24 h (37°C, 5% CO_2_), visual observation of the culture per well and plating of cultures in petri dishes with brain heart infusion (BHI) agar (Himedia, Dindhori, Nashik, India) were performed. A 10-μL aliquot of each undiluted culture was used for plating with BHI agar. After that, 100 μL of these cultures was removed from each well and transferred to microtubes containing 900 μL of 0.89% NaCl (Química Moderna, Barueri, SP, Brazil) for serial dilution and plating on BHI agar. Plates were incubated (48 h, 37°C, 5% CO_2_), followed by colony counting. Data CFU were transformed into log_10_ and analyzed.

### S. mutans cells recovered from biofilms posttreatment.

The inoculum of the bacterial strain was prepared as described above. For this, 22 μL of the mid-log culture (OD_540_ of 0.847 ± 0.273) was added to a Falcon tube containing 14.978 mL of TY culture medium with 1% (wt/vol) sucrose (Synth, Diadema, SP, Brazil). This dilution was performed to standardize the culture at 1 × 10^6^ CFU/mL. After vortexing, a 100-μL aliquot was distributed to each well of five 96-well polystyrene microplates (Falcon, Corning, NY, USA). These plates were incubated (1.5 h, 37°C, 5% CO_2_), allowing bacterial cell adhesion to the well’s bottom. Each plate used had a layout to contain two consecutive treatment cycles (in duplicate). From the biofilm inoculum, a 100-μL aliquot was transferred to microtubes containing 900 μL of 0.89% NaCl, followed by serial dilution, plating, incubation (48 h, 37°C, 5% CO_2_), and colony counting (data for the inoculum control across experiments).

After bacterial adhesion (1.5 h), nonadhered cells were removed by aspirating the culture medium volume from each well, followed by washing with 100 μL of 0.89% NaCl. Then, the agents were added to microtubes containing TY plus 1% sucrose (plus vehicle—in adequate volumes of diluents to achieve 7% EtOH and 1.25% DMSO) and later transferred to their respective wells in the microplates.

The wells corresponding to the adhesion control were washed twice with 100 μL of 0.89% NaCl, and 200 μL of 0.89% NaCl was added per well. Next, these wells were processed, with three consecutive washes and scraping, as follows: 200 μL of 0.89% NaCl was removed by scraping the bottom of the well, whose contents were transferred to an empty 1.5-mL microtube. Next, 200 μL of 0.89% NaCl was added (per well), and the scraping process and volume transfer to the corresponding microtube were repeated. The process was repeated using an additional 100 μL of 0.89% NaCl, yielding 500 μL of the undiluted or pure suspension of adhered cells. These suspensions were homogenized by vortexing for 30 s and were serially diluted and plated on BHI agar. These plates were incubated (48 h, 37°C, 5% CO_2_), and colony counting was performed. Next, the five 96-well microplates were incubated (20 h, 37°C, 5% CO_2_).

After 20 h of incubation, the culture medium was removed by aspirating the culture medium from each well, followed by visual observation of growth—isolated colonies or biofilm on the well’s bottom. Next, the wells corresponding to cycle 1 were washed twice with 200 μL of 0.89% NaCl, leaving 200 μL of 0.89% NaCl in them. Then, treatments and controls were added to each well. The wells corresponding to cycle 1 were processed as described for the adhesion control wells, and the 96-well microplates were incubated (20 h, 37°C, 5% CO_2_).

These procedures (addition of treatments, incubation, removal of the culture media, visual observation, washing of the wells from the previous cycle, treatment of plates, and processing of the cycle) were repeated until the processing of cycle 10 (21). Then, CFU data were transformed into log_10_ and analyzed. After the colony count data were obtained, colonies were frozen in 50% glycerol (100 μL/colony) and used for the next steps (Table 1). Three colonies from each selected cycle were frozen for each agent on each experimental occasion. The selected cycle was the one before the absence of colony growth on agar plates (see Fig. S1 in the supplemental material).

### Biofilm formation on saliva-coated hydroxyapatite discs for characterization.

S. mutans UA159 cells recovered posttreatment were used to grow biofilms on saliva-coated hydroxyapatite (sHA) discs (diameter, 12 mm; thickness, 1 mm) (Clarkson Chromatography Products, Inc., South Williamsport, PA, USA) in batch cultures for 67 h, as detailed previously (8). Saliva and pellicles were prepared as described before (60). Saliva was donated by three volunteers who had not had antimicrobial treatments in the last 3 months. Each volunteer masticated a piece of Parafilm (Parafilm M; Sigma-Aldrich Co., St. Louis, MO) and collected saliva into an ice-chilled Falcon tube. The saliva samples from all volunteers were pooled and diluted 1:1 with adsorption buffer (AB buffer) (50 mM KCl, 1 mM KPO_4_, 1 mM CaCl_2_, 1 mM MgCl_2_, 0.1 mM phenylmethylsulfonyl fluoride [PMSF] in double-distilled water [ddH_2_O; pH 6.5]). Saliva was centrifuged (8, 60) at 3,220 × *g* for 20 min at 4°C in an Eppendorf centrifuge 5810R, and the clarified portion was filtered sterilized with a 75-mm-diameter, 500-mL Rapid-Flow, 0.2-μm-pore aPES (polyethersulfone) membrane filter (Nalgene, Thermo Scientific, Mexico). Saliva was used fresh for pellicle formation and medium preparation at the start of the experiment. Any remaining saliva was aliquoted and stored at −80°C for the subsequent culture medium preparation. Saliva-coated HA discs were placed in a vertical position in three 24-well plates (Kasvi, Beijing, China) using a custom-made disc holder (14).

S. mutans cells recovered from posttreatment cultures were grown to the mid-exponential growth phase and were diluted in TY plus 0.1% sucrose and 20% saliva until they reached 1 × 10^6^ CFU/mL. The biofilms were incubated for 19 h (37°C, 5% CO_2_) (Fig. S2).

The culture media were changed twice daily: at 19 h and 43 h (TY plus 0.1% sucrose and 20% saliva) and at 29 h and 53 h (0.5% sucrose plus 1% starch [Sigma-Aldrich Co., St. Louis, MO] and 25% saliva) (8, 60). After each change of medium, the pH of the spent medium was measured. The biofilms were grown and analyzed in two developmental phases: 45 h for gene expression assessment and 67 h for microbial population, insoluble dry weight (biomass), protein content in the insoluble portion of biofilms, biochemical characteristics of biofilm extracellular matrix (in the soluble and insoluble portions), and confocal microscopy (architecture) analyses.

### (i) Gene expression analysis.

At 45 h of growth, biofilms were removed and processed for total RNA isolation. First, the biofilms were dip-washed into wells containing sterilized 0.89% NaCl solution. Each biofilm (disc) was transferred to a glass tube. Next, 1 mL of RNAlater stabilization solution (Ambion, Molecular Probes, Austin, TX, USA) was used to wash the walls of each tube. The glass tubes with biofilms/discs were placed in a beaker and subjected to water bath sonication for 11 min for biofilm detachment from discs. A sterile metal spatula was used to scrape any remaining biofilm from each disc. Each biofilm suspension was transferred to a new Falcon tube, and 1 mL of RNAlater was used to rinse the glass and the discs; this volume was recovered and stored in the corresponding Falcon tubes, totaling 2 mL of biofilm suspension. The biofilm suspensions were stored at −80°C until RNA isolation.

The selected S. mutans genes were associated with insoluble exopolysaccharides (*gtfB*), eDNA (*lrgA*), LTA (*dltB*), tolerance to acid (*atpD* and *nox1*) and oxidative stresses (*nox1*), and glycolysis (*eno*, encoding enolase enzyme) (61, 62). In addition, three genes were evaluated for data normalization (*16S rRNA, gyrA*, and *recA*).

RNA was isolated according to the methodology optimized for biofilms (63) through the phenol-chloroform separation method and purified via treatment with DNase in a column (RNeasy microkit; Qiagen, Austin, TX, USA) and in solution (Turbo DNase; Ambion, Austin, TX, USA). DNase was removed using the RNeasy MinElute cleanup kit (Qiagen). After that, spectrophotometry was used to evaluate the quantity (in nanograms per microliter (OD_260_) and purity (OD_260/280_ ratio) of RNA samples (Nano-spectrophotometer DS-11+; Denovix)). The yield was calculated depending on the volume in which the RNA was diluted. The integrity of the purified RNA was verified by 1% agarose gel electrophoresis (Ultra Pure; Invitrogen).

cDNA synthesis was performed in duplicate per sample using 0.5 μg of total RNA and the iScript kit (Bio-Rad Laboratories, Inc., Hercules, CA, USA, catalog no. 170-8890). Negative controls were prepared without using reverse transcriptase to determine whether there was DNA contamination. Reaction mixtures were incubated using the CFX96 Touch real-time PCR detection system (Bio-Rad), with the following cycles: 25°C for 5 min, 42°C for 30 min, 85°C for 5 min, and 4°C to ∞. The cDNA samples were stored at −20°C until used to quantify gene expression (quantitative PCR [qPCR]).

qPCR analyses were carried out using specific primers (Table 3). cDNA was diluted 1:5 for specific genes and 1:1,000 for the *16S rRNA* gene. cDNA negative controls were not diluted. cDNA and negative controls were amplified by a CFX96 system (Bio-Rad) using specific primers (Table 3) and iQ SYBR green supermix (Bio-Rad). A standard curve based on the PCR product was plotted for each primer set (64). The reactions were run using the following cycles with CFX96 system (Bio-Rad) equipment: 95°C for 3 min, followed by 35 cycles of 94°C for 15 min, 58°C for 30 min, 68°C for 15 min, 95°C for 1 min, 55°C for 1 min, and then 80 cycles of 55°C for 10 min. The standard curves were used to transform the quantification cycle (*C_q_*) values to relative numbers of cDNA molecules. Next, the expression data were analyzed via fold change using the vehicle control group as a reference.

**TABLE 3:** Primers used for RT-qPCR

Gene	GenBank locus tag	Sequence^*a*^	Primer concn (nM)	Product size (bp)	Reference
*16S rRNA*		ACCAGAAAGGGACGGCTAAC	200	122	41
		TAGCCTTTTACTCCAGACTTTCCTG			
*gtfB*	SMU_1004	AAACAACCGAAGCTGATAC	250	90	41
		CAATTTCTTTTACATTGGGAAG			
*nox1*	SMU_765	GGACAAGAATCTGGTGTTGA	250	91	61
		CAATATCAGTCTCTACCTTAGGC			
*atpD*	SMU_1528c	GGCGACAAGTCTCAAAGAATTG	250	115	61
		AACCATCAGTTGACTCCATAGC			
*lrgA*	SMU_575c	GTCTATCTATGCTGCTATT	300	109	62
		AAGGACATACATGAGAAC			
*dltB*	SMU_1690	TGTCTTCATGTCTATCTTAC	250	130	62
		TTCCATAATCATACCTACTG			
*eno*	SMU_1247	GTTGAACTTCGCGATGGAGAT	250	150	This study
		GTCAAGTGCGATCATTGCTTTAT			
*recA*	SMU_1114	GCGTGCCTTGAAGTTTTATTCTTC	250	75	67
		TGTTCCCCGGTTCCTTTAATT			
*gyrA*	SMU_2085	ATTGTTGCTCGGGCTCTTCCAG	250	105	68
		ATGCGGCTTGTCAGGAGTAACC			

a For each pair of sequences, the forward sequence is on the top and the reverse sequence is on the bottom.

### (ii) Biofilm analyses to determine viable population, dry weight, protein, and matrix composition.

At 67 h of development, biofilms were processed for analyses following previously described protocols (8, 60). Briefly, biofilms were dip-washed into wells containing sterile 0.89% NaCl. Each biofilm (disc) was transferred to a glass tube containing 1 mL of 0.89% NaCl. Next, 1 mL of 0.89% NaCl was used to wash the walls of each tube. The glass tubes with biofilms/discs were placed in a beaker containing distilled water and subjected to water bath sonication (CD4820; Kondortech Digital, São Carlos, SP, Brazil) for 11 min. A sterile metal spatula was used to scrape any remaining biofilm from each disc (8), and 2 mL of each biofilm suspension was transferred to a new 15-mL Falcon tube. Next, each glass tube was washed with 3 mL of 0.89% NaCl, which was transferred to the tube containing the initial 2 mL, yielding a 5-mL total biofilm suspension per biofilm/disc.

Each biofilm suspension (5 mL) was sonicated using a probe at 7 W for 30 s (Sonicator model Q125; QSonica, Newtown, CT, USA). An aliquot of each suspension (0.1 mL) was used for a serial dilution and plating on BHI agar (37°C, 5% CO_2_, 48 h) to determine CFU. The remaining volume (4.9 mL) was centrifuged (3,220 × *g*, 20 min, 4°C). The supernatant (with soluble extracellular matrix components) was transferred to a new tube, and the pellet (precipitate with the microbial cells and insoluble matrix components) was washed twice with sterile Milli-Q water at 2.6 and 2.5 mL (3,220 × *g*, 20 min, 4°C). The supernatants generated during the two washes were combined with the first supernatant obtained, totaling 10 mL, which was used to isolate and quantify water-soluble exopolysaccharides (6 mL) (60, 65), eDNA (0.5 mL) (8), protein content (in the soluble portion of the matrix) (0.5 mL) (8) and LTA (3 mL) (8). The pellet was suspended in 1.0 mL of Milli-Q water, from which 10 μL was used to determine the protein content (in the insoluble portion of biofilms), and 0.99 mL was used for quantification of dry weight (insoluble biomass), followed by the isolation and quantification of water-insoluble exopolysaccharides (or alkali-soluble polysaccharides) (60, 65).

### (iii) Confocal laser scanning microscopy imaging.

Biofilms were formed and treated as described above, except that Alexa Fluor 647-labeled dextran conjugate (absorbance/fluorescence emission maxima of 647/668 nm; Molecular Probes, Carlsbad, CA, USA) was added to the culture medium at the beginning of biofilm formation (0 h) and each culture medium change (19 h, 29 h, 43 h, and 53 h) (66). This strategy enables the incorporation of labeled dextrans into exopolysaccharides during their synthesis and matrix buildup. When the biofilms reached 67 h of development, the discs were dip-washed into wells containing 0.89% NaCl and transferred to wells containing 0.89% NaCl and SYTO9 (485/498 nm; Molecular Probes), which is a green fluorescent nucleic acid marker for visualization of bacteria.

The imaging of the three-dimensional structure of these biofilms was performed using a Zeiss LSM 800 microscope (Zeiss, Jena, Germany) fitted with a 20× lens objective. Each biofilm was scanned at least in two randomly selected positions, and a series of confocal images were generated by optical sectioning at each of these positions with a Zeiss LSM800, with Multialkali-PMT detector, 488-nm (SYTO9) and 561-nm (Alexa Fluor 647) laser, 20× EC Plan-Neofluar objective, 0.312- by 0.312-μm scale per pixel, and 1.5-μm increments. The images were analyzed using ZEN Blue 2.3 software for 3D reconstruction of exopolysaccharides and bacteria. Furthermore, each image was analyzed using COMSTAT (version 2) software for quantification of total bacterial content and exopolysaccharide matrix (biovolume) and the percentage of coverage per area from the interface substrate/biofilm (HA disc) to the top (outer layer) of each biofilm (41).

### Statistical analyses.

The data obtained were submitted to descriptive and inferential statistical analyses to compare groups (agents and controls used). Data were organized in a database (Excel) and analyzed with statistical tests according to distribution. The Prism 9 statistical software (GraphPad Software, Inc., San Diego, CA, USA) was used, with a significance level of 5%. For distribution analysis, the Shapiro-Wilk test was used. As the sample was small and a well-controlled laboratory test was used, a parametric test was used: one- or two-way ANOVA followed by Tukey’s posttest. Also, a qualitative analysis of confocal images was performed.

Data from killing curves and cells recovered posttreatment were submitted to only descriptive statistics and analyzed longitudinally. Biofilm characterization data were submitted to descriptive and inferential statistics. They included the following response variables: biomass (milligrams), proteins (micrograms), population (CFU per biofilm), extracellular matrix components (insoluble exopolysaccharides [micrograms], soluble exopolysaccharides [micrograms], proteins [micrograms], LTA [micrograms], and eDNA [micrograms]), and gene expression (gene copies per microliter). Also, the quantification of the three-dimensional structures (bacteria and exopolysaccharides) included as response variables biovolume (cubic micrometers/square micrometers), maximum thickness (square micrometers), and the percentage of area occupied by the biofilm layer.
