# Supplementary material for: Distinct Agents Induce Streptococcus mutans Cells with Altered Biofilm Formation Capacity
Source: Microbiol Spectr. 2022 Jul 11;10(4):e00650-22. doi: 10.1128/spectrum.00650-22 (PMC9430944; doi:10.1128/spectrum.00650-22)
Supplement: Supplemental file 1 — Fig. S1 to S3; Tables S1 to S4. Download spectrum.00650-22-s0001.pdf, PDF file, 0.3 MB [file spectrum.00650-22-s0001.pdf]

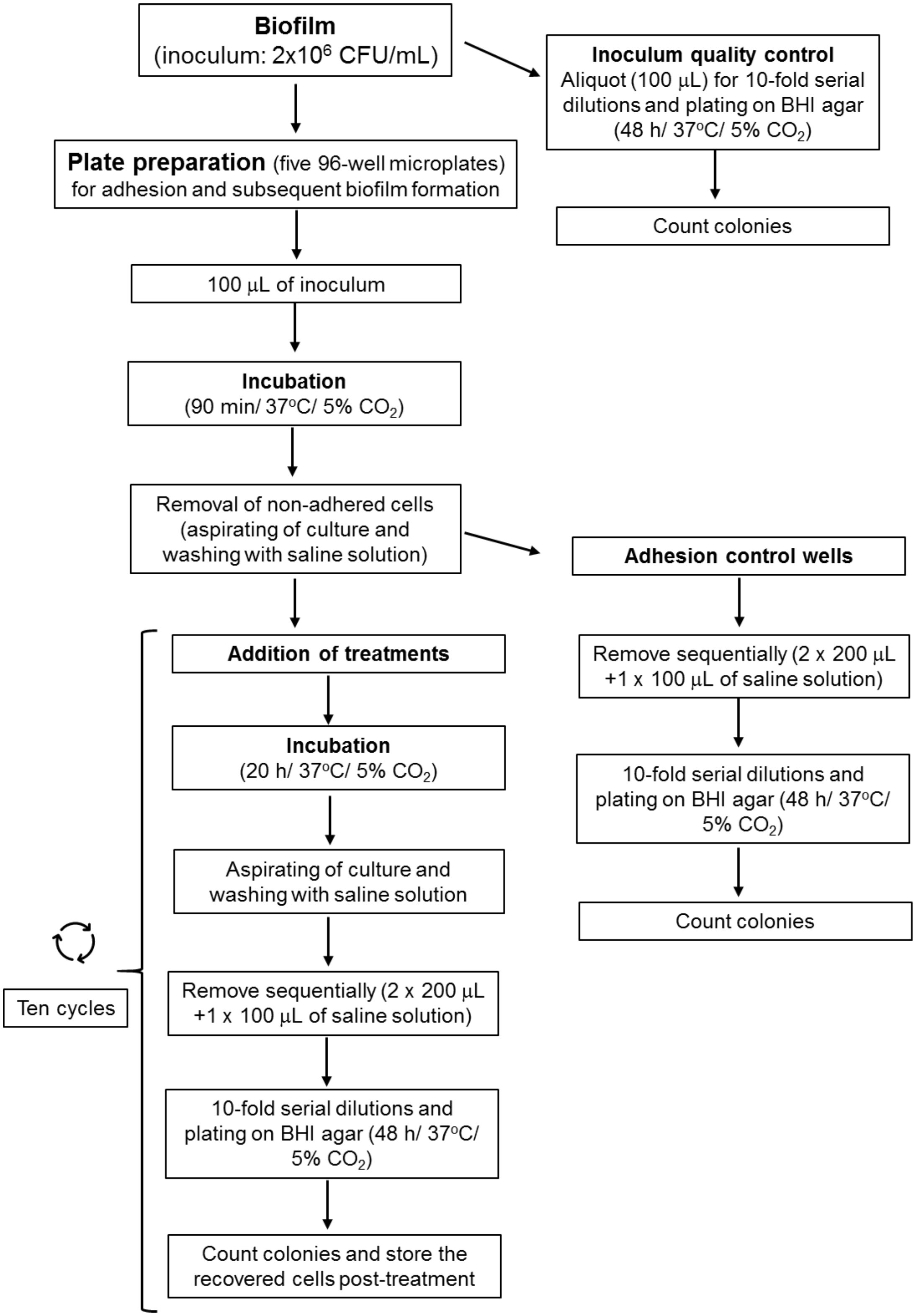

Figure S1. ***S. mutans*** **recovered cells post-treatment of biofilms.** The sequence of procedures performed to recover cells that persisted post-treatment using distinct agents and controls.

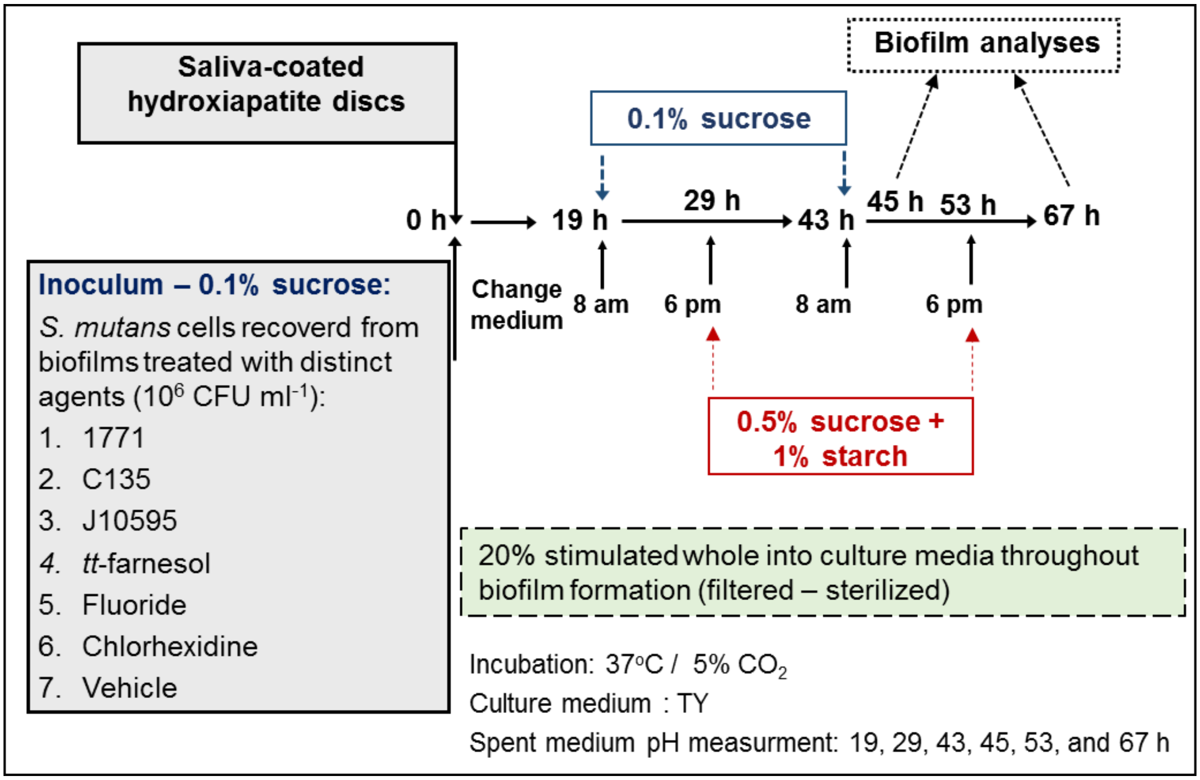

Figure S2. Experimental design of biofilms on hydroxyapatite discs formed by *S. mutans* cells recovered post-treatment with distinct agents and controls.

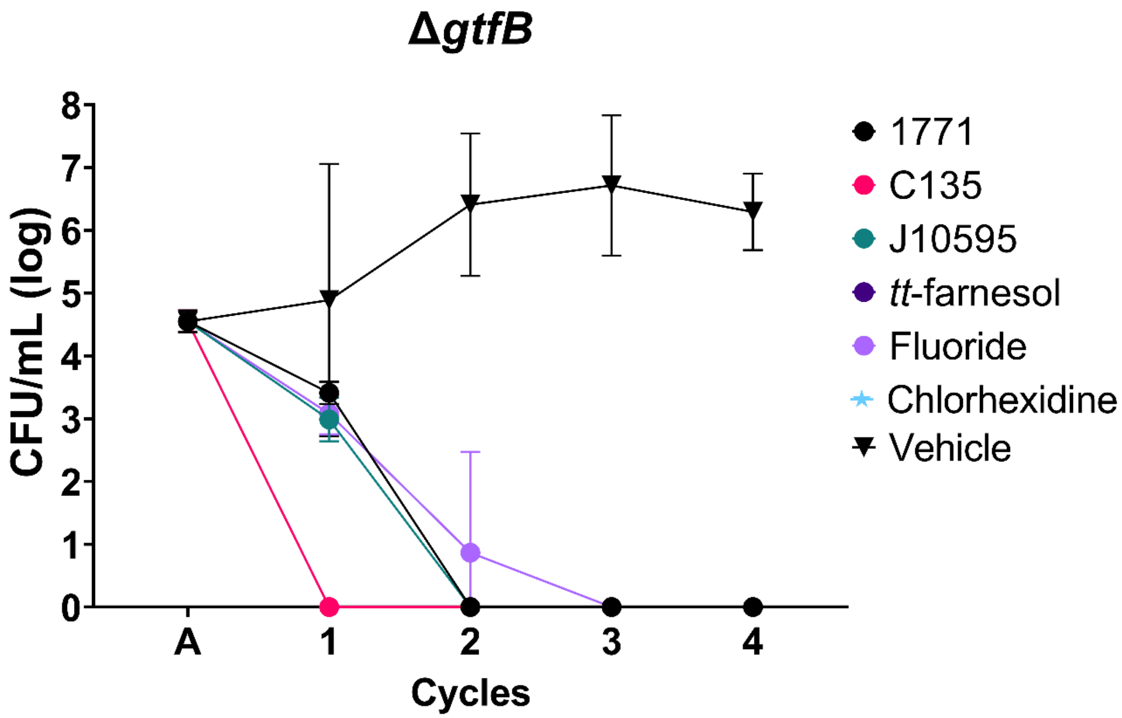

Figure S3. Δ*gtfB* cells recovered post-treatment using distinct agents and controls. Each time is represented by the mean, and error bars are the standard deviation. 1771: compound 1771; C135: 4’ hydroxychalcone; J10595: myricetin. A: adhesion.

Table S1. ***Streptococcus mutans*** **growth inhibition (TY+1% glucose) and killing curves (TY) by agents incubated with cells at mid-log and stationary growth phases**. Data shown are the mean (± standard deviation or SD) of CFU/mL (without log10 transformation). Data are from three experiments performed in duplicate per experiment.

Medium and growth phase		Time	CFU/mL						
			1771	C135	J10595	*tt*-farnesol	Fluoride	Chlorexidine	V
TY+1% glucose	mid-log	0 h	5.08x10^{6} (± 5.10x10^{6})	5.08x10^{6} (± 5.10x10^{6})	5.08x10^{6} (± 5.10x10^{6})	5.08x10^{6} (± 5.10x10^{6})	5.08x10^{6} (± 5.10x10^{6})	5.08x10^{6} (± 5.10x10^{6})	5.08x10^{6} (± 5.10x10^{6})
		1 h	2.74x10^{6} (± 2.02x10^{6})	0	1.68x10^{6} (± 9.59x10^{5})	1.26x10^{3} (± 5.18x10^{2})	2.08x10^{6} (± 1.99x10^{6})	0	1.70x10^{6} (± 1.77x10^{6})
		2 h	2.64x10^{6} (± 1.88x10^{6})	0	1.12x10^{6} (± 4.97x10^{5})	6.92x10^{2} (± 8.81x10^{2})	2.89x10^{6} (± 2.36x10^{6})	0	2.14x10^{6} (± 2.31x10^{6})
		3 h	2.67x10^{6} (± 1.86x10^{6})	0	6.81x10^{5} (± 6.16x10^{5})	5.33x10^{2} (± 4.62x10^{2})	2.31x10^{6} (± 1.75x10^{6})	0	2.08x10^{6} (± 1.98x10^{6})
		4 h	2.73x10^{6} (± 2.10x10^{6})	0	4.19x10^{5} (± 2.83x10^{5})	2.33x10^{2} (± 2.42x10^{2})	2.43x10^{6} (± 1.48x10^{6})	0	2.31x10^{6} (± 2.18x10^{6})
		6 h	3.56x10^{6} (± 1.59x10^{6})	0	2.00x10^{5} (± 2.11x10^{5})	1.89x10^{2} (± 3.52x10^{2})	3.48x10^{6} (± 1.21x10^{6})	0	2.36x10^{6} (± 2.28x10^{6})
		24 h	4.91x10^{5} (± 4.93x10^{4})	3.02x10^{7} (± 5.51x10^{7})	0	0	2.96x10^{5} (± 2.71x10^{5})	0	2.91x10^{8} (±1.86 x10^{8})
	stationary	0 h	1.72x10^{6} (± 1.46x10^{6})	1.72x10^{6} (± 1.46x10^{6})	1.72x10^{6} (± 1.46x10^{6})	1.72x10^{6} (± 1.46x10^{6})	1.72x10^{6} (± 1.46x10^{6})	1.72x10^{6} (± 1.46x10^{6})	1.72x10^{6} (± 1.46x10^{6})
		1 h	9.35x10^{5} (± 3.47x10^{5})	0	8.60x10^{5} (± 3.03x10^{5})	1.19x10^{2} (± 2.08x10^{2})	9.50x10^{5} (± 3.19x10^{5})	0	1.36x10^{6} (± 1.78x10^{6})
		2 h	8.95x10^{5} (± 2.5x10^{5})	0	6.10x10^{5} (± 2.69x10^{5})	5.71x10^{1} (± 9.97x10^{1})	8.50x10^{5} (± 3.07x10^{5})	0	1.07x10^{6} (± 1.45x10^{6})
		3 h	7.66x10^{5} (± 3.13x10^{5})	0	6.10x10^{5} (± 3.16x10^{5})	0	9.00x10^{5} (± 3.43x10^{5})	0	1.28x10^{6} (± 1.65x10^{6})
		4 h	8.00x10^{5} (± 2.78x10^{5})	0	5.88x10^{5} (± 2.33x10^{5})	0	6.55x10^{5} (± 2.52x10^{5})	0	1.18x10^{6} (± 1.51x10^{6})

		6 h	$1.15x10^6$ (± $1.07x10^6$)	0	$1.19x10^6$ (± $1.58x10^6$)	0	$1.93x10^6$ (± $1.59x10^6$)	0	$9.42x10^5$ (± $7.89x10^5$)
		24 h	$1.27x10^4$ (± $8.16x10^3$)	$9.95x10^4$ (2.72± $x10^5$)	$5.67x10^2$ (± $1.03x10^3$)	0	$6.52x10^4$ (± $6.49x10^4$)	0	$2.28x10^8$ (± $1.78x10^8$)
TY	mid-log	0 h	$4.72x10^5$ (± $2.57x10^5$)	$4.72x10^5$ (± $2.57x10^5$)	$4.72x10^5$ (± $2.57x10^5$)	$4.72x10^5$ (± $2.57x10^5$)	$4.72x10^5$ (± $2.57x10^5$)	$4.72x10^5$ (± $2.57x10^5$)	$4.72x10^5$ (± $2.57x10^5$)
		1 h	$3.57x10^5$ (± $7.18x10^4$)	$5.31x10^4$ (± 8.06 $x10^4$)	$3.02x10^5$ (± $1.03x10^5$)	$2.80x10^5$ (± $1.25x10^5$)	$2.24x10^5$ (± $5.70x10^4$)	0	$3.63x10^5$ (± $7.38x10^4$)
		2 h	$3.16x10^5$ (± 6.67 $x10^4$)	$7.02x10^2$ (± $1.53x10^3$)	$2.30x10^5$ (± $5.06x10^4$)	$1.12x10^4$ (± $8.61x10^3$)	$3.25x10^5$ (± $4.04x10^4$)	0	$3.86x10^5$ (± $1.18x10^5$)
		3 h	$3.22x10^5$ (± 9.31 $x10^4$)	$6.90x10^2$ (± $1.41x10^3$)	$2.31x10^5$ (± $6.89x10^4$)	$4.58x10^3$ (± $3.89x10^3$)	$3.08x10^5$ (± $6.76x10^4$)	0	$3.45x10^5$ (± $6.86x10^4$)
		4 h	$3.38x10^5$ (± $1.10x10^5$)	$5.10x10^2$ (± 1.08 $x10^3$)	$1.99x10^5$ (± $9.24x10^4$)	$2.20x10^3$ (± $1.36x10^3$)	$2.79x10^5$ (± $5.44x10^4$)	0	$3.76x10^5$ (± $7.12x10^4$)
		6 h	$2.63x10^5$ (± 1.11 $x10^5$)	$4.10x10^2$ (± 8.67 $x10^2$)	$1.92x10^4$ (± $7.40x10^3$)	$3.13x10^2$ (± $3.04x10^2$)	$2.96x10^5$ (± 5.45 $x10^4$)	0	$1.06x10^6$ (± $1.57x10^6$)
		24 h	$1.36x10^4$ (± $6.91x10^3$)	$1.50x10^2$ (± $3.81x10^2$)	$8.35x10^3$ (± $1.17x10^4$)	0	$2.74x10^4$ (± $2.10x10^4$)	0	$1.94x10^7$ (± $1.58x10^7$)
	stationary	0 h	$3.63x10^5$ (± $2.65x10^5$)	$3.63x10^5$ (± $2.65x10^5$)	$3.63x10^5$ (± $2.65x10^5$)	$3.63x10^5$ (± $2.65x10^5$)	$3.63x10^5$ (± $2.65x10^5$)	$3.63x10^5$ (± $2.65x10^5$)	$3.63x10^5$ (± $2.65x10^5$)
		1 h	$3.93x10^5$ (± $7.05x10^4$)	$4.05x10^4$ (± $4.74x10^4$)	$4.80x10^5$ (± $1.07x10^5$)	$2.18x10^5$ (± $1.13x10^5$)	$4.02x10^5$ (± $9.12x10^4$)	0	$3.85x10^5$ (± $4.52x10^4$)
		2 h	$3.77x10^5$ (± $5.43x10^4$)	$1.06x10^4$ (± $1.47x10^4$)	$3.48x10^5$ (± $5.02x10^4$)	$6.17x10^3$ (± $5.40x10^3$)	$4.03x10^5$ (± $5.74x10^4$)	0	$3.67x10^5$ (± $3.73x10^4$)
		3 h	$3.45x10^5$ (± $4.06x10^4$)	$1.04x10^4$ (± $1.47x10^4$)	$3.50x10^5$ (± $7.22x10^4$)	$2.37x10^3$ (± $1.60x10^3$)	$3.52x10^5$ (± $9.57x10^4$)	0	$3.74x10^5$ (± $5.47x10^4$)
		4 h	$3.53x10^5$ (± $4.56x10^4$)	$7.45x10^3$ (± $9.97x10^3$)	$2.92x10^5$ (± $5.95x10^4$)	$2.49x10^3$ (± $3.16x10^3$)	$3.67x10^5$ (± $6.89x10^4$)	0	$3.27x10^5$ (± $5.02x10^4$)
		6 h	$2.72x10^5$ (± $4.71x10^4$)	$7.47x10^3$ (± $9.74x10^3$)	$2.27x10^5$ (± $3.98x10^4$)	$1.03x10^3$ (± $1.22x10^3$)	$3.18x10^5$ (± $7.24x10^4$)	0	$8.77x10^5$ (± $1.34x10^6$)
		24 h	$1.40x10^4$ (± $7.62x10^3$)	$2.78x10^3$ (± $3.38x10^3$)	$4.33x10^2$ (± $3.89x10^2$)	$2.50x10^1$ (± $4.52x10^1$)	$4.05x10^4$ (± $5.11x10^3$)	0	$2.04x10^7$ (± $1.40x10^7$)

Table S2. *S. mutans* cells recovered post-treatment. Data shown are the mean (± standard deviation or SD) of CFU/mL (log). Data are from three experiments performed in duplicate per experiment.

Adhesion and treatment cycles	CFU/mL (log)						
	1771	C135	J10595	*tt*-farnesol	Fluoride	Chlorexidine	V
Adhesion*	4.34 (±0.25)	4.34 (±0.25)	4.34 (±0.25)	4.34 (±0.25)	4.34 (±0.25)	4.34 (±0.25)	4.34 (±0.25)
1	4.05 (±0.56)	1.65 (±2.26)	3.45 (±0.32)	1.29 (±2.04)	3.07 (±1.48)	1.86 (±2.11)	7.55 (±0.11)
2	3.83 (±1.20)	3.87 (±3.27)	1.38 (±1.75)	2.32 (±3.09)	1.17 (±1.74)	0.65 (±1.51)	7.12 (±0.36)
3	4.36 (±0.25)	3.99 (±2.96)	0.52 (±1.21)	0.00 (±0.00)	0.58 (±1.04)	0.54 (±1.27)	6.79 (±0.43)
4	4.25 (±0.20)	3.39 (±3.54)	0.00 (±0.00)	0.00 (±0.00)	0.00 (±0.00)	0.19 (±0.66)	6.85 (±0.19)
5	3.66 (±1.85)	2.00 (±2.98)	0.00 (±0.00)	1.10 (±2.57)	0.00 (±0.00)	0.53 (±1.25)	6.66 (±0.10)
6	4.59 (±0.80)	3.68 (±3.50)	0.19 (±0.66)	1.99 (±2.79)	0.00 (±0.00)	0.00 (±0.00)	6.78 (±0.17)
7	4.87 (±1.11)	6.32 (±0.32)	0.00 (±0.00)	1.88 (±2.78)	0.00 (±0.00)	0.75 (±1.76)	6.57 (±0.27)
8	4.86 (±1.24)	6.78 (±0.17)	0.47 (±1.11)	1.07 (±2.06)	0.42 (±0.99)	1.30 (±1.93)	6.73 (±0.15)
9	5.42 (±0.47)	1.61 (±2.99)	0.00 (±0.00)	0.00 (±0.00)	0.00 (±0.00)	0.92 (±1.71)	6.48 (±0.23)
10	5.86 (±0.39)	6.10 (±0.53)	0.00 (±0.00)	0.00 (±0.00)	0.00 (±0.00)	0.78 (±1.44)	6.72 (±0.10)

*Adhesion: adhesion present the same values for all treatments because these are the cells that were adhered to the surfaces before introducing agents.

Table S3. ***S. mutans* cells recovered post-treatment.** Data shown are the mean (± standard deviation or SD) of CFU/mL (without log10 transformation). Data are from three experiments performed in duplicate per experiment.

Adhesion and treatment cycles	CFU/mL						
	1771	C135	J10595	*tt*-farnesol	Fluoride	Chlorexidine	V
Adhesion*	$2.53x10^4$ ($\pm1.66x10^4$)	$2.53x10^4$ ($\pm1.66x10^4$)	$2.53x10^4$ ($\pm1.66x10^4$)	$2.53x10^4$ ($\pm1.66x10^4$)	$2.53x10^4$ ($\pm1.66x10^4$)	$2.53x10^4$ ($\pm1.66x10^4$)	$2.53x10^4$ ($\pm1.66x10^4$)
1	$2.91x10^4$ ($\pm4.92x10^4$)	$8.02x10^4$ ($\pm1.88x10^4$)	$3.62x10^3$ ($\pm2.66x10^3$)	$2.17x10^4$ ($\pm5.08x10^4$)	$5.87x10^3$ ($\pm6.14x10^3$)	$2.10x10^4$ ($\pm4.71x10^4$)	$3.63x10^7$ ($\pm1.04x10^7$)
2	$7.80x10^4$ ($\pm1.12x10^5$)	$4.23x10^6$ ($\pm5.25x10^6$)	$1.45x10^3$ ($\pm2.58x10^3$)	$2.76x10^6$ ($\pm6.34x10^6$)	$1.47x10^3$ ($\pm2.80x10^3$)	$1.27x10^3$ ($\pm2.96x10^3$)	$1.88x10^7$ ($\pm1.88x10^7$)
3	$2.64x10^4$ ($\pm1.48x10^4$)	$8.33x10^5$ ($\pm9.15x10^5$)	$2.17x10^2$ ($\pm5.08x10^2$)	0	$5.00x10^1$ ($\pm9.05x10^1$)	$3.17x10^2$ ($\pm7.70x10^2$)	$9.87x10^6$ ($\pm1.03x10^7$)
4	$1.94x10^4$ ($\pm8.36x10^3$)	$3.02x10^6$ ($\pm3.21x10^6$)	0	0	0	$1.67x10^1$ ($\pm5.77x10^1$)	$7.77x10^6$ ($\pm3.73x10^6$)
5	$5.64x10^4$ ($\pm7.68x10^4$)	$6.50x10^6$ ($\pm1.38x10^6$)	0	$6.83x10^5$ ($\pm1.61x10^6$)	0	$2.67x10^2$ ($\pm6.29x10^2$)	$4.68x10^6$ ($\pm1.13x10^6$)
6	$1.02x10^5$ ($\pm1.34x10^5$)	$5.33x10^6$ ($\pm7.55x10^6$)	$1.67x10^1$ ($\pm5.77x10^1$)	$1.97x10^6$ ($\pm4.66x10^6$)	0	0	$6.37x10^6$ ($\pm2.37x10^6$)
7	$4.38x10^5$ ($\pm5.82x10^5$)	$2.67x10^6$ ($\pm2.02x10^6$)	0	$1.83x10^5$ ($\pm3.46x10^5$)	0	$5.50x10^3$ ($\pm1.30x10^3$)	$4.40x10^6$ ($\pm2.70x10^6$)
8	$4.66x10^5$ ($\pm5.73x10^5$)	$6.37x10^6$ ($\pm2.12x10^6$)	$1.17x10^2$ ($\pm2.76x10^2$)	$3.00x10^4$ ($\pm7.01x10^4$)	$6.67x10^1$ ($\pm1.78x10^2$)	$2.98x10^3$ ($\pm5.04x10^3$)	$5.75x10^6$ ($2.33\pm x10^6$)
9	$4.13x10^5$ ($\pm3.94x10^5$)	$7.25x10^5$ ($\pm1.37x10^6$)	0	0	0	$1.25x10^3$ ($\pm2.38x10^3$)	$3.43x10^6$ ($\pm1.84x10^6$)
10	$9.90x10^5$ ($\pm7.20x10^5$)	$2.08x10^6$ ($\pm1.69x10^6$)	0	0	0	$3.25x10^2$ ($\pm6.23x10^2$)	$5.38x10^6$ ($\pm1.25x10^6$)

Table S4. Viable counts of *S. mutans* of 67 h-old biofilms derived from cells recovered post-treatments. Data shown are the mean (± standard deviation or SD) of CFU/mL (without log10 transformation). Data are from three experiments performed in duplicate per experiment.

CFU/mL						
1771	C135	J10595	*tt*-farnesol	Fluoride	Chlorexidine	V
$3.05x10^{8}$ (± $1.44x10^{8}$)	$3.04x10^{8}$ (±$2.01x10^{8}$)	$3.35x10^{8}$ (±$2.41x10^{8}$)	$2.69x10^{8}$ (±$1.40x10^{8}$)	2.92 $x10^{8}$ (±$9.19x10^{7}$)	$1.27x10^{8}$ (±$6.04x10^{7}$)	$4.19x10^{8}$ (±$2.90x10^{8}$)
